# Development of Li_4_SiO_4_ sorbents from Argentine diatomite for high-temperature CO_2_ capture

**DOI:** 10.1039/d6ra05886h

**Published:** 2026-09-07

**Authors:** Eider David Torres, María Laura Grasso, Fabiana Gennari, Pierre Arneodo Larochette

**Affiliations:** a National University of Cuyo-Balseiro Institute Av. Bustillo 9500 S. C. de Bariloche Río Negro R8402AGP Argentina fabiana.gennari@ib.edu.ar; b Bariloche Atomic Center-National Atomic Energy Commission (CAB-CNEA) Av. Bustillo 9500 S. C. de Bariloche Río Negro R8402AGP Argentina; c National Council for Scientific and Technical Research (CONICET) Av. Bustillo 9500 S. C. de Bariloche Río Negro R8402AGP Argentina

## Abstract

Although Li_4_SiO_4_-based sorbents show great potential for high-temperature CO_2_ capture, conventional formulations derived from pure SiO_2_ suffer from high synthesis costs and kinetic limitations. This work proposes a sustainable alternative by utilizing raw, unpurified natural diatomite from Río Negro (Argentina) as the silicon precursor. A series of Li_4_SiO_4_ sorbents were prepared *via* a two-step mechano-thermal method, optimizing the initial *n*Li_2_CO_3_–SiO_2_ molar ratio to 1.7–1.8 to maximize yield and performance. Physicochemical, thermodynamic, and kinetic properties were evaluated and contrasted against a commercial reagent reference (LS). The diatomite-derived sorbent (LD) achieved remarkable experimental CO_2_ capture capacities of 27 wt% under pure CO_2_ (at 650 °C) and 22 wt% under a diluted 10% CO_2_ atmosphere (at 575 °C) demonstrating excellent cyclic stability over 10 cycles. Thermodynamic and kinetic studies *via* the van 't Hoff and Kissinger equations yielded a reaction enthalpy of 135 kJ mol^−1^ and a decarbonation activation energy of 210 kJ mol^−1^, outperforming the commercial benchmark. Structural and morphological analyses suggest that native impurities in the diatomite likely induce a beneficial lattice contraction, while promoting the *in situ* formation of a stable LiFeO_2_ secondary phase alongside an alkali-metal eutectic molten phase. Rather than hindering performance, these built-in dopants act synergistically to prevent sintering and bypass solid-state diffusion barriers. These findings validate Argentine diatomite as a strategically advantageous, low-cost regional precursor for manufacturing highly efficient Li_4_SiO_4_ sorbents tailored for practical carbon capture application.

## Abstract 2

Li_4_SiO_4_ synthesized from Argentine diatomite exhibits efficient, reversible CO_2_ capture at lower temperatures than a commercial reference, even under 10% CO_2_, combining preserved thermodynamics with improved sorption kinetics

## Graphical abstract

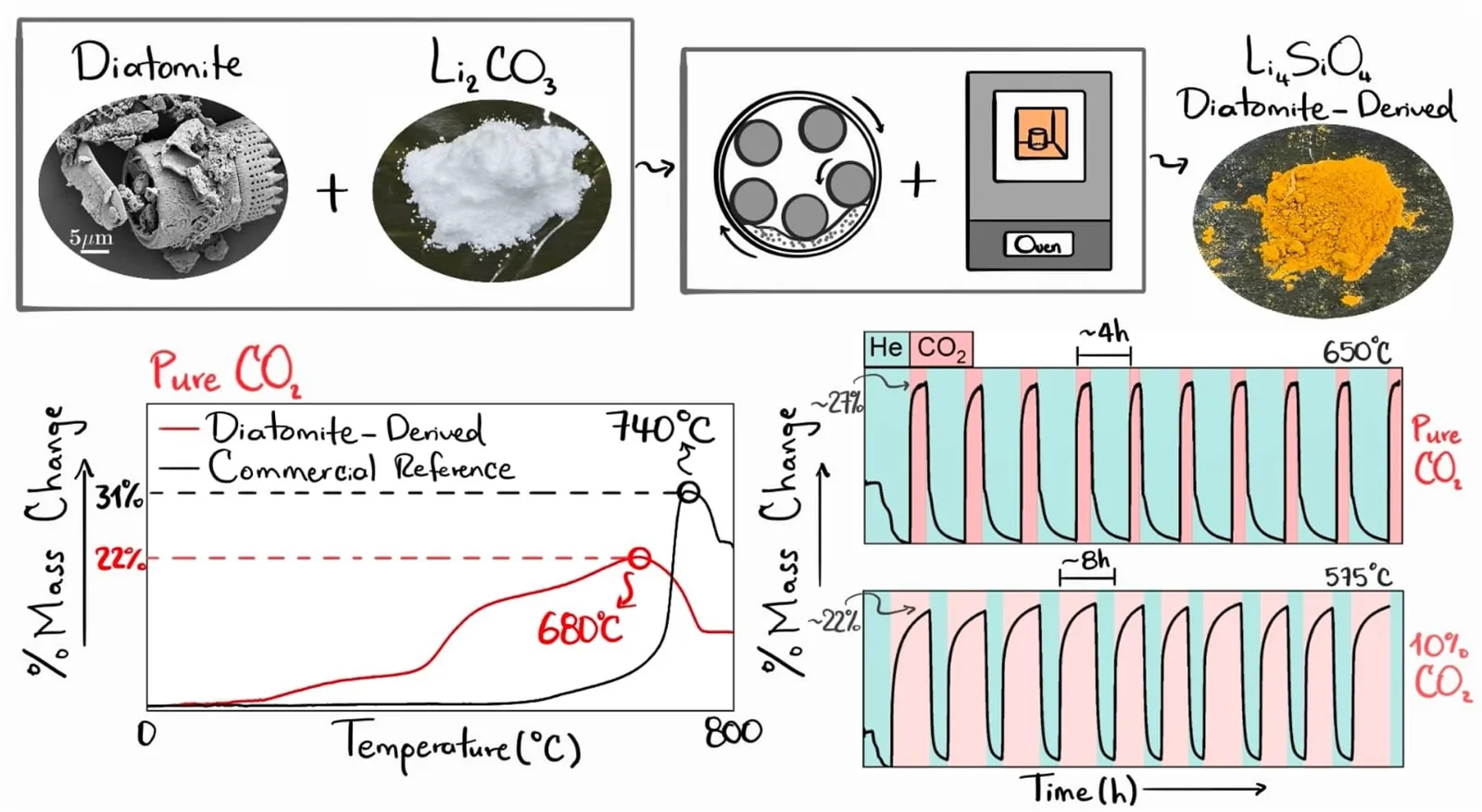

## 1 Introduction

The accelerating rate of anthropogenic climate change is directly linked to the unprecedented accumulation of greenhouse gases, with carbon dioxide (CO_2_) accounting for approximately 81% of the increase in total radiative forcing over the last decade.^1^ Driven by continuous fossil fuel combustion, atmospheric CO_2_ reached a historic global average of 423.9 ± 0.2 ppm after experiencing an unprecedented annual surge of 3.5 ppm.^1^ This alarming trajectory is intrinsically tied to global energy consumption, which continues to expand to meet modern industrial demands. Despite the rapid incorporation of renewable energy sources into the global matrix, the sheer magnitude of this demand has outpaced green transitions, leaving fossil power in a dominant position.^2,3^ Consequently, mitigating continuous emissions from fossil fuel sources has become one of the most urgent challenges of our century, driving the development of advanced engineering strategies to capture CO_2_ directly at its source and stabilize the global climate.^4,5^

To mitigate these pressing environmental impacts, Carbon Capture and Storage (CCS) technologies have been recognized as one of the most critical and efficient strategies for reducing CO_2_ emissions directly from large-scale industrial point sources.^4,5^ Currently, these technological frameworks are categorized into pre-combustion capture, post-combustion capture, and oxy-fuel combustion approaches.^5,6^ Within pre-combustion processes, methods such as sorption-enhanced steam methane reforming (SESMR) simultaneously capture CO_2_ and produce high-purity hydrogen, highlighting the versatility of these systems.^7^ A key factor in optimizing the efficiency and economic viability of these operations involves the use of high-temperature solid sorbents, which can directly separate CO_2_ from flue or process gases without the energy penalty associated with cooling the streams.^8,9^ Consequently, identifying and developing high-temperature chemical sorbents that exhibit both an excellent sorption capacity and robust cyclic stability under realistic operational conditions has become a primary focus of contemporary research.^9,10^

Among the various high-temperature solid sorbents investigated to date, CaO-based materials have been extensively studied due to its rapid kinetics and substantial initial absorption capacity at temperatures around 550–700 °C.^11,12^ However, CaO-based sorbents suffer from severe sintering during the high-temperature desorption phase (>900 °C), causing their cyclic capacity to decay drastically after multiple operations.^13^ In comparison, lithium orthosilicate (Li_4_SiO_4_) has shown many advantages^14–16^ including high theoretical sorption capacity (36.7 wt%) based on the reaction (1), satisfactory sorption kinetics and superior thermal cyclability:

1 Li_4_SiO_4_ (s) + CO_2_ (g) ↔ Li_2_SiO_3_ (s) + Li_2_CO_3_ (s)

Despite these advantages, the application of pristine Li_4_SiO_4_ can still face certain kinetic limitations depending on its synthesis-induced structural properties. To overcome these intrinsic bottlenecks, extensive research has established that CO_2_ capture performance can be heavily enhanced introducing additives to accelerate carbonation kinetics. Generally, two of the most effective doping strategies include: (i) alkali metal doping to form eutectic carbonates that reduce CO_2_ diffusion resistance;^17–19^ (ii) transition or alkaline-earth metal doping to substitute cations in the lattice, generating vacancies that facilitate ion diffusion.^20–23^ While these compositional modifications are conventionally achieved using expensive, high-purity chemical reagents through complex synthesis routes, identifying cost-effective alternative precursors has emerged as a critical requirement for industrial scalability.

To address this challenge, recent academic efforts have turned toward naturally occurring silicon-containing minerals as alternative precursors. Beyond standard synthetic silica (such as amorphous silica gel, fumed silica, or quartz powder), natural minerals like diatomite, halloysite, perlite and vermiculite have already demonstrated outstanding results, often yielding adequate CO_2_ sorption capacity and promising cyclic stability.^24–27^ Crucially, these low-cost mineral precursors inherently contain natural impurities. Rather than acting as detrimental defects, these multi-element impurities could potentially substitute cations in the Li_4_SiO_4_ framework or induce the formation of beneficial *in situ* eutectic phases during thermal treatment. Consequently, natural minerals could mimic advanced synthetic doping strategies without the economic burden of chemical-grade additives.

In this work, we explore the preliminary potential of a natural diatomite sourced from Río Negro, Argentina, as a promising and sustainable silicon precursor for Li_4_SiO_4_ synthesis. Due to its unique geological origin, this specific diatomite naturally incorporates a variety of elements that can act as multi-functional, built-in dopants. Consequently, this raw material offers a synergistic advantage, potentially modifying the final microstructure and accelerating reaction rates without the need for expensive synthetic doping procedures.

Herein, the Li_4_SiO_4_ sorbent was prepared *via* a two-step mechano-thermal process, and the synthesis parameters were preliminary optimized to maximize its CO_2_ absorption capacity. Comprehensive thermodynamic and kinetic studies were performed to compare preliminary performance of the diatomite-derived sorbent against a counterpart synthesized from commercial-grade reagents. Specifically, the influence of the mineral's secondary phases and native impurities on the carbonation kinetics and cyclic stability was investigated under two CO_2_ partial pressures. Ultimately, this study aims to demonstrate a cost-effective and baseline pathway for developing functional lithium-silicate sorbents with potential for intermediate-temperature carbon capture applications.

## 2 Experimental

### 2.1. Synthesis of Li_4_SiO_4_-based sorbents from diatomite

The starting materials used were commercial lithium carbonate (Li_2_CO_3_, Cicarelli, 99% purity) and diatomite as a silicon source (natural resource from Río Negro, Argentina). The natural silicon source was sieved to obtain a fraction with a particle size equal to or less than 0.3 mm. The elemental composition of the diatomite was determined by EDS and AAS (Table 1). To obtain statistically representative bulk composition data from EDS, measurements were conducted on compacted powder samples by averaging more than 10 independent area scans across several square micrometers. High agreement is observed for major elements (Si, Mg, Ca, Al, Fe). The lower Na content obtained by AAS (0.01 wt%) compared to bulk-representative EDS (1.6 wt%) is attributed to incomplete extraction of alkali species trapped within the refractory silicate matrix during acid digestion.

**Table 1:** Elemental composition (in wt%) of the diatomite from Río Negro (Argentina) determined by EDS and AAS

Element	Na	Mg	Al	Si	K	Ca	Fe	Ti	Others
EDS^a^	1.6	1.2	10.8	70.4	2.9	2.4	6.6	0.7	3.4
AAS	0.01	1.3	13.7	70.2	4.5	2.3	8.0	—	—

a EDS values represent the mean of >10 large-area scans of 75 µm × 45 µm, performed on compacted powder pellets to ensure statistical bulk representativeness.

The synthesis of lithium orthosilicate (Li_4_SiO_4_) sorbents was carried out by a two-step process,^28^ which involve milling of the *n*Li_2_CO_3_–SiO_2_ mixture followed by annealing at a defined temperature, according to the equation:

2 2 Li_2_CO_3_ (s) + SiO_2_ (s) → Li_4_SiO_4_ (s) + 2 CO_2_ (g)

Different *n*Li_2_CO_3_–SiO_2_ precursor mixtures were prepared with molar ratios of 1.5 : 1, 1.7 : 1, 1.8 : 1, 2 : 1 (stoichiometry of eqn (2)), and 2.2 : 1. To ensure adequate homogenization, all mixtures were ball-milled in a Fritsch Pulverisette P6 planetary mill for 1 hour at room temperature under an air atmosphere. Stainless steel milling chambers and balls were utilized with a ball-to-sample weight ratio of 50. Milling was conducted at a rotational speed of 400 rpm, implementing 10-minute cooling breaks every 15 minutes of grinding to prevent sample overheating.

The synthesis temperature of 600 °C was selected based on literature reports^28,29^ and our preliminary investigations. Specifically, to evaluate the thermal behavior and confirm this reaction temperature, a combined TG-DSC analysis (Linseis STA PT-1600) of the ball-milled mixture with a 1.7 : 1 molar ratio was performed under an air flow (50 sccm) from room temperature to 600 °C at a heating rate of 5 °C min^−1^ (see Section 3.1). Following the thermal treatment at 600 °C, the resulting materials are hereafter referred to as Li_4_SiO_4_-based sorbents. For clarity, two specific formulations are highlighted:

- Sorbent synthesized from natural diatomite with an optimal initial *n*Li_2_CO_3_–SiO_2_ molar ratio of 1.7 : 1 is designated as LD.

- As reference, two benchmark samples were prepared using commercial Li_2_CO_3_ and SiO_2_ (Sigma-Aldrich, analytical grade) in a stoichiometric 2 : 1 molar ratio. This mixture was processed under identical milling conditions and calcined at 600 °C for 24 h and 800 °C for 5 h.^28^ The resulting commercial silica-derived sorbent are denoted as LS600 and LS800, respectively.

### 2.2. Estimation of theoretical CO_2_ capture capacity of Li_4_SiO_4_

The theoretical CO_2_ capture capacity for each composite was calculated based on a two-step solid-state reaction mechanism. First, the lithium orthosilicate (Li_4_SiO_4_) is synthesized from lithium carbonate (Li_2_CO_3_) and silicon dioxide (SiO_2_) according to eqn (2). Subsequently, the carbonation reaction responsible for CO_2_ capture is governed by the reaction (1). To standardize the calculations for each initial molar ratio (*R* = *n*Li_2_CO_3_/SiO_2_), a baseline of 1 mol of SiO_2_ was established (*n*SiO_2_ = 1), considering two operational scenarios based on the limiting reagent:

(1) For *R* < 2 (Li_2_CO_3_ limited): the moles of formed Li_4_SiO_4_ and remaining unreacted were determined by *n*(Li_4_SiO_4_)=R/2 and *n*(SiO_2_)_excess_ = 1 − R/2, respectively.

(2) For *R* ≥ 2 (SiO_2_ limited): full conversion of SiO_2_ was assumed (*n*(Li_4_SiO_4_) = 1), leaving an excess of unreacted Li_2_CO_3_ (*n*(Li_2_CO_3_)_excess_ = *R* − 2).

The pure sorbent mass after calcination (*m*_sorb, pure_) and the corresponding mass of captured CO_2_ were computed using the respective molar masses.

To account for the chemical composition of the natural silicon source, a purity correction factor was introduced. The natural diatomite utilized contains 78 wt% of active SiO_2_ determined by calculating the elemental oxide percentages based on their most stable oxidized phases. Consequently, an unreacted inert mass fraction (*m*_inert_ ∼16.95 g per mole of pure SiO_2_) was incorporated into the final sorbent weight. The corrected theoretical capacity (*C*_real_, wt%) was thus determined as follows:3
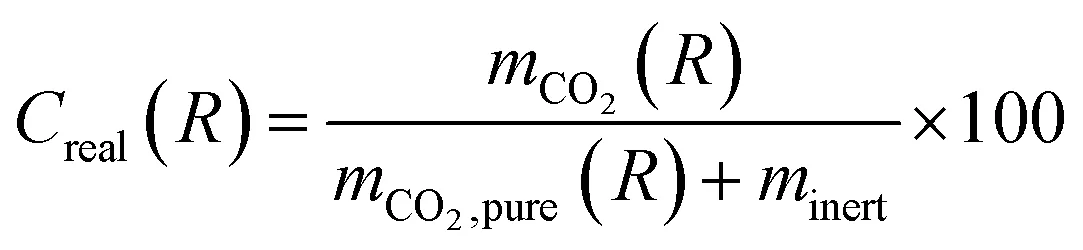


The resulting corrected theoretical capture values for the studied compositions (1.5 : 1 to 2.2 : 1) range between 27 wt% and 32 wt% CO_2_.

### 2.3. Characterization of Li_4_SiO_4_ and analysis of CO_2_ capture properties

The structural, textural, thermal, and morphological characteristics of the reagents, milled mixtures (*n*Li_2_CO_3_–SiO_2_), and post-reaction samples were investigated using X-ray powder diffraction (XRD), TG-DSC (Thermogravimetry-Differential Scanning Calorimetry), N_2_ soprtion isotherms and SEM-EDS (Scanning Electron Microscopy combined with Energy Dispersive X-ray Spectroscopy). XRD patterns were recorded on a Bruker D8 Advance diffractometer (CuKα radiation), over a 2*θ* range of 10–80°, with a step size of 0.015° and a dwell time of 0.75 seconds. Using XRD data and the Scherrer equation the crystallite size was estimated. Simultaneous TG-DSC measurements (Linseis STA PT-1600) were performed to evaluate thermal stability and sorbent performance. Tests were conducted at a heating rate of 5 °C min^−1^ under either air (50 sccm) or 10°C min^−1^ under dynamic 10% or 100% of CO_2_ flows (50 sccm). The resulting mass change (%) was expressed as mass percentage relative to the initial sample mass. Sample morphology and composition were examined *via* SEM-EDS (Zeiss Crossbeam 340). Prior to analysis, samples were dispersed on carbon tape and gold-sputter coated. N_2_ isotherms were measured at −196 °C (Micromeritics ASAP 2020) after overnight degassing at 350 °C (0.2 g). Surface area and pore distribution were determined *via* the BET method.

The CO_2_ capture properties of the sorbents were evaluated using a high-pressure thermogravimetric system (TG-HP50, TA Instruments). For each test, approximately 30–40 mg of sample was placed in an alumina crucible. Four complementary types of TG measurements were performed to quantify the CO_2_ capture capacity and determine the thermodynamic and kinetic parameters of the carbonation reaction:

(i) CO_2_ capture capacity: the system was initially heated at 10 °C min^−1^ from room temperature to the target temperature under He atmosphere with a total flow rate of 30 sccm. Subsequently, the gas flow was switched to 30 sccm either pure CO_2_ or 10% of CO_2_ (inert gas balance), while maintaining the temperature and pressure constant at selected values. The resulting mass change (%) was used to estimate the maximum capture capacity at the selected temperature, expressed as mass percentage relative to the decarbonated sample mass.

(ii) CO_2_ absorption/desorption cycling: following the aforementioned initialization procedure and upon reaching a quasi-steady-state mass (maximum capacity), the atmosphere was cyclically alternated between an inert gas (He) and CO_2_ at constant temperature. Isothermal sorption stages were carried out using pure CO_2_ or 10% CO_2_ (inert gas balance). The absorption segments were considered complete when the mass change rate fell below 3 × 10^−5^ mg s^−1^. For the desorption stages, the inlet gas was switched to He to achieve an inert atmosphere within the reactor at the same temperature, maintaining these conditions until desorption was complete. These cycles served to evaluate the operational stability, reversibility, and capacity retention of the sorbents over time.

(iii) Pressure-composition isotherm (PCI) measurements: the sample was heated under a He flow (30 sccm) at atmospheric pressure until the target temperature was reached and then allowed to stabilize for 30 min. Subsequently, the pressure was reduced to the mechanical-pump base pressure while the gas was switched to a low CO_2_ flow (3–5 sccm). The pressure was then increased at a rate of 0.02 torr s^−1^ up to 500 torr (1000 torr for the base material), while continuously recording the mass change. This low pressurization rate allowed the system to remain close to equilibrium throughout the measurement.

From these isotherms, the equilibrium pressure at each temperature was determined and the reaction enthalpy (Δ*H*_r_) was calculated using the van 't Hoff equation.

(iv) Activation energy: to determine the activation energy of the carbonation (decarbonation) reaction, non-isothermal experiments were conducted at multiple heating rates (*β*) under a CO_2_ (Ar) atmosphere. The peak temperatures (*T*_p_) of the absorption or desorption events were identified from the derivative thermogravimetric (DTG) curves. Using the corresponding heating rates and *T*_p_ values, the activation energy (*E*_a_) was calculated *via* the Kissinger method.^30^

## 3 Results and discussion

### 3.1. Synthesis of Li_4_SiO_4_ using diatomite as Si source

The synthesis of Li_4_SiO_4_ was carried out using Li_2_CO_3_ and diatomite as starting materials, according to reaction (2). In the first stage, the Li_2_CO_3_-diatomite mixture was subjected to mechanical milling to achieve a homogeneous blend of the precursor materials. In the second stage, the reagent mixture was thermally treated at 600 °C. This synthesis temperature was selected based on the thermal behavior of the milled Li_2_CO_3_–SiO_2_ mixture (1.7 : 1 molar ratio) during heating in air (Fig. S1). The TG-DSC curves reveal two distinct mass loss steps correlated with endothermic events. The initial mass loss (up to 150 °C) of approximately 8 wt% is attributed to sample dehydration. A second, more pronounced mass loss of about 33 wt% occurs at higher temperatures and ceases after a 2 h isothermal dwell at 600 °C. This second event is ascribed to the decomposition of Li_2_CO_3_, which is promoted by the presence of the acidic silicon oxides from the diatomite.

To determine whether the formation of Li_4_SiO_4_ was completed within this 2 h interval, XRD studies were performed. Fig. 1 presents the X-ray diffraction patterns of the starting diatomite and Li_2_CO_3_ (Fig. 1a and b, respectively), as well as the mixture after ball milling (Fig. 1c) and subsequent thermal treatment at 600 °C for 2 h (Fig. 1d). In the raw diatomite precursor, the following crystalline phases were identified: SiO_2_ (quartz; ICDD 01-078-1252), hydrotalcite (ICDD 01-089-0460, (Mg_0.66_Al_0.33_)(OH)_2_(CO_3_)_0.17_(H_2_O)_0.5_), muscovite (ICDD 00-007-0025, KAl_2_Si_3_AlO_10_(OH)_2_), and albite (ICDD 01-078-1995, NaAlSi_3_O_8_). This indicates that the silicon content (Table 1) is distributed across different mineral structures, which may exhibit differential chemical reactivity. In the milled mixture (Fig. 1c), the primary crystalline phase corresponds to the starting Li_2_CO_3_, alongside residual quartz and albite.

**Fig. 1:**
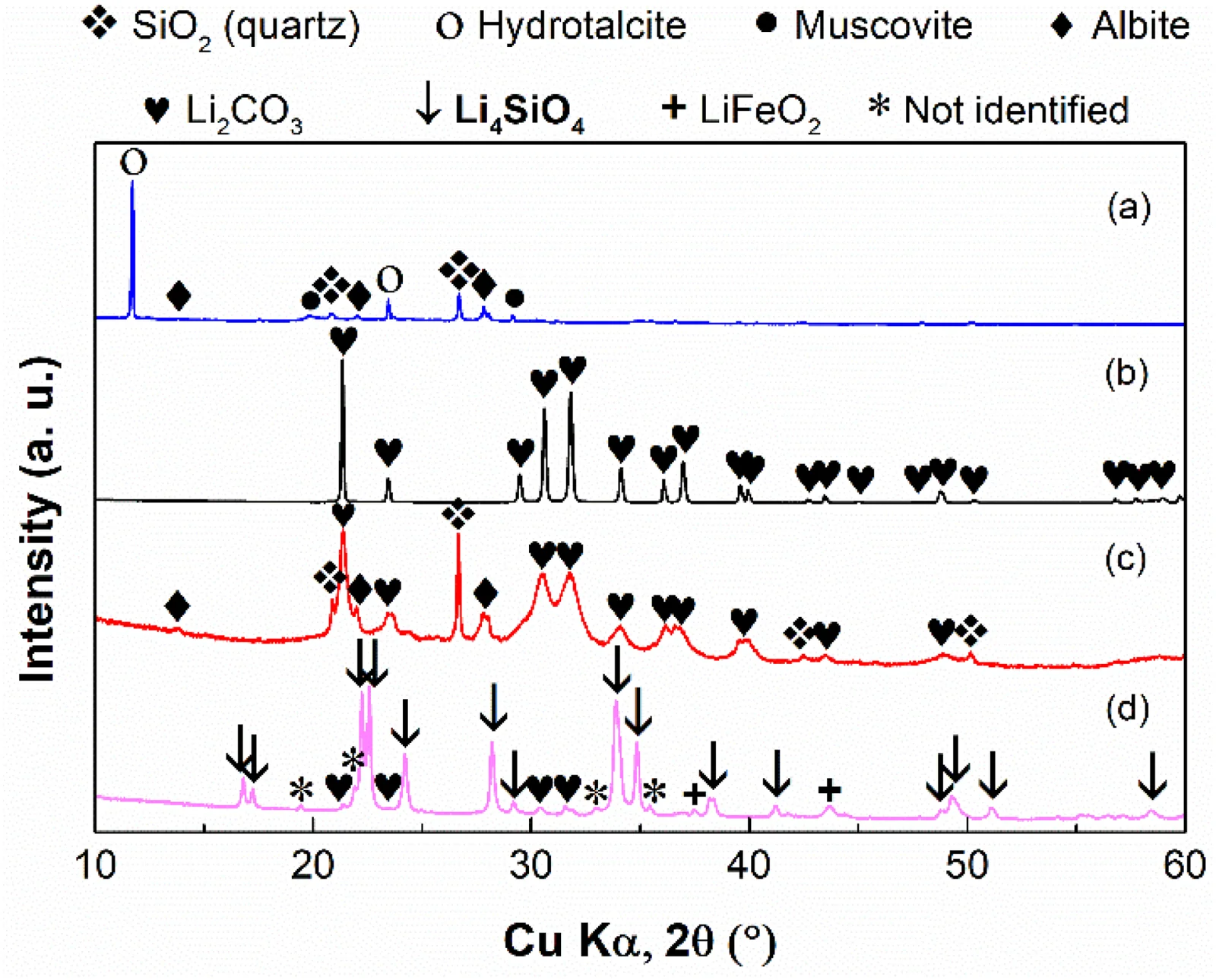
XRD patterns of (a) diatomite, (b) Li_2_CO_3_, (c) 1.7 Li_2_CO_3_–SiO_2_ milled mixture, and (d) sample (c) thermal treated at 600 °C for 2 h.

Following the thermal treatment (Fig. 1d), the formation of the target Li_4_SiO_4_ product (ICDD 01-076-1085) was confirmed. However, unreacted Li_2_CO_3_ was also detected, indicating that the reaction between the precursors was incomplete under these conditions. Concurrently, minor amounts of LiFeO_2_ (ICDD 01-089-7118) and an unidentified secondary phase were observed. These secondary phases likely originate from impurities inherently present in the natural diatomite used as the silicon source.

Given that Li_4_SiO_4_ was formed as the majority phase but unreacted lithium precursor remained, two distinct strategies were implemented to optimize the synthesis. First, the thermal treatment at 600 °C was extended to 24 h to promote the solid-state reaction between the reagents. Second, considering that the initial precursor homogeneity and composition are crucial for maximizing the Li_4_SiO_4_ yield, and consequently, enhancing its CO_2_ capture capacity, the effect of the initial *n*Li_2_CO_3_–SiO_2_ molar ratio was systematically evaluated.

To achieve this objective, precursor mixtures with different molar ratios (1.5 : 1, 1.7 : 1, 1.8 : 1, 2 : 1, and 2.2 : 1) were milled and subsequently heated at 600 °C for 24 h. The XRD analysis of these samples after the thermal treatment (Fig. 2) reveals how variations in the initial *n*Li_2_CO_3_–SiO_2_ molar ratio affect the yield of the active Li_4_SiO_4_ phase. As the ratio increases beyond 1.7 : 1, the diffraction peaks associated with Li_4_SiO_4_ become less defined, whereas the reflections corresponding to unreacted Li_2_CO_3_ progressively intensify. Notably, for 1.7 : 1 and 1.8 : 1 molar ratios, Li_2_CO_3_ reflections are virtually tracking close to the detection limit or absent in the patterns, suggesting a complete reaction of the lithium precursor under these specific conditions. Furthermore, a secondary phase corresponding to Li_2_CaSiO_4_ (ICDD 01-072-1729) emerges at higher molar ratios. Indeed, the inherent impurities and amorphous nature of the natural diatomite alter the reaction pathways compared to the benchmark commercial silica reference.

**Fig. 2:**
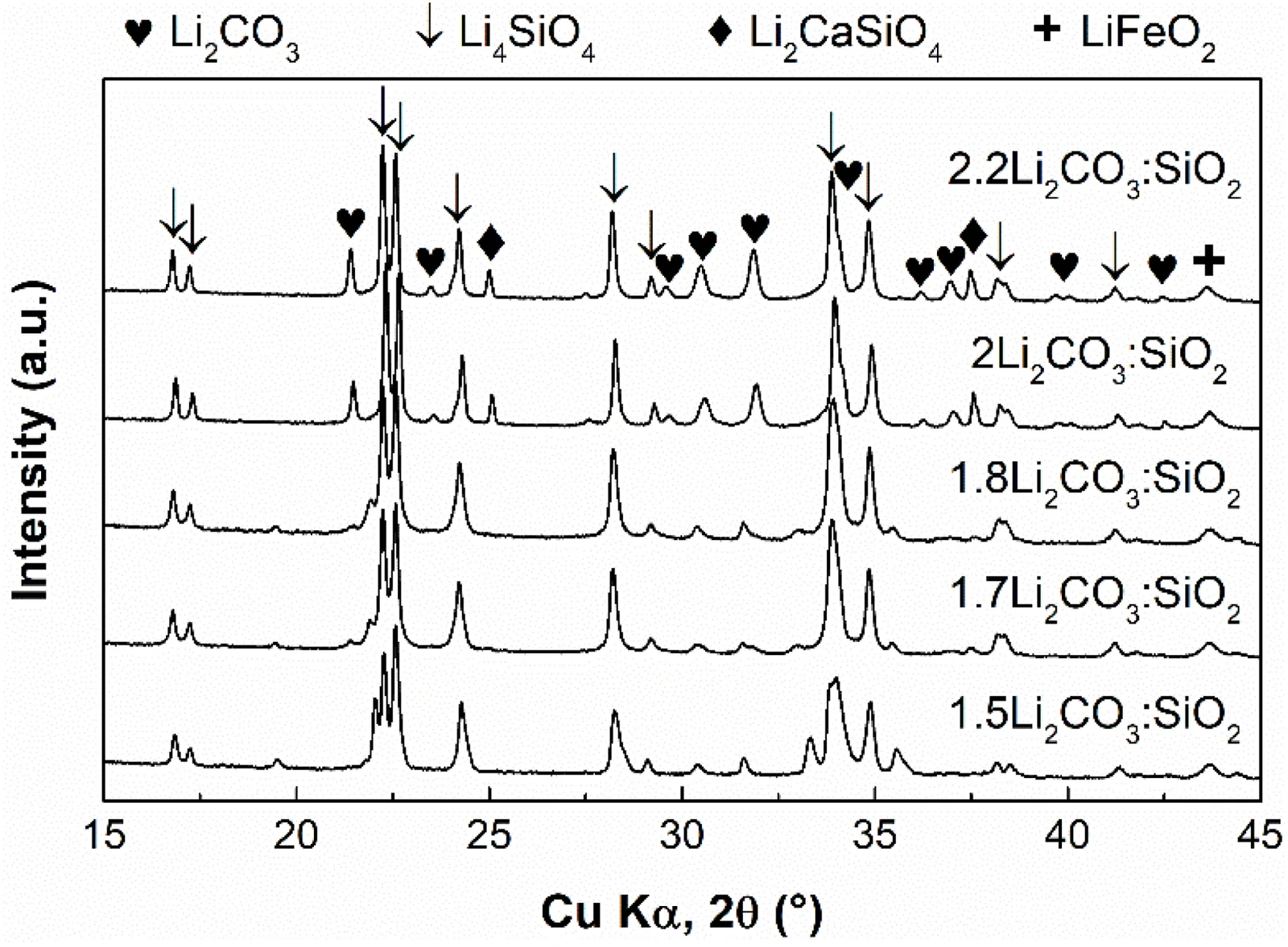
XRD patterns of the Li_4_SiO_4_-based sorbents synthesized at 600 °C for 24 h from ball milled *n*Li_2_CO_3_–SiO_2_ mixtures with various molar ratios.

To determine the optimal ratio for subsequent CO_2_ capture performance, the Li_4_SiO_4_-based sorbents obtained by varying the *n*Li_2_CO_3_–SiO_2_ molar ratios were preheated under an inert atmosphere in the TG system up to 650 °C. At which point the gas flow was switched to CO_2_ (1 bar) to measure the maximum CO_2_ uptake (Fig. S2). As shown in Fig. 3, the capture capacity exhibits a maximum for *n*Li_2_CO_3_–SiO_2_ molar ratios between 1.7 : 1 and 1.8 : 1. This maximum coincides with the molar ratio where unreacted Li_2_CO_3_ was practically undetected. Consequently, the 1.7 : 1 ratio was selected as optimal, as it maximizes the CO_2_ capture capacity *via* Li_4_SiO_4_ carbonation while minimizing secondary phases, such as the excess unreacted Li_2_CO_3_ reagent.

**Fig. 3:**
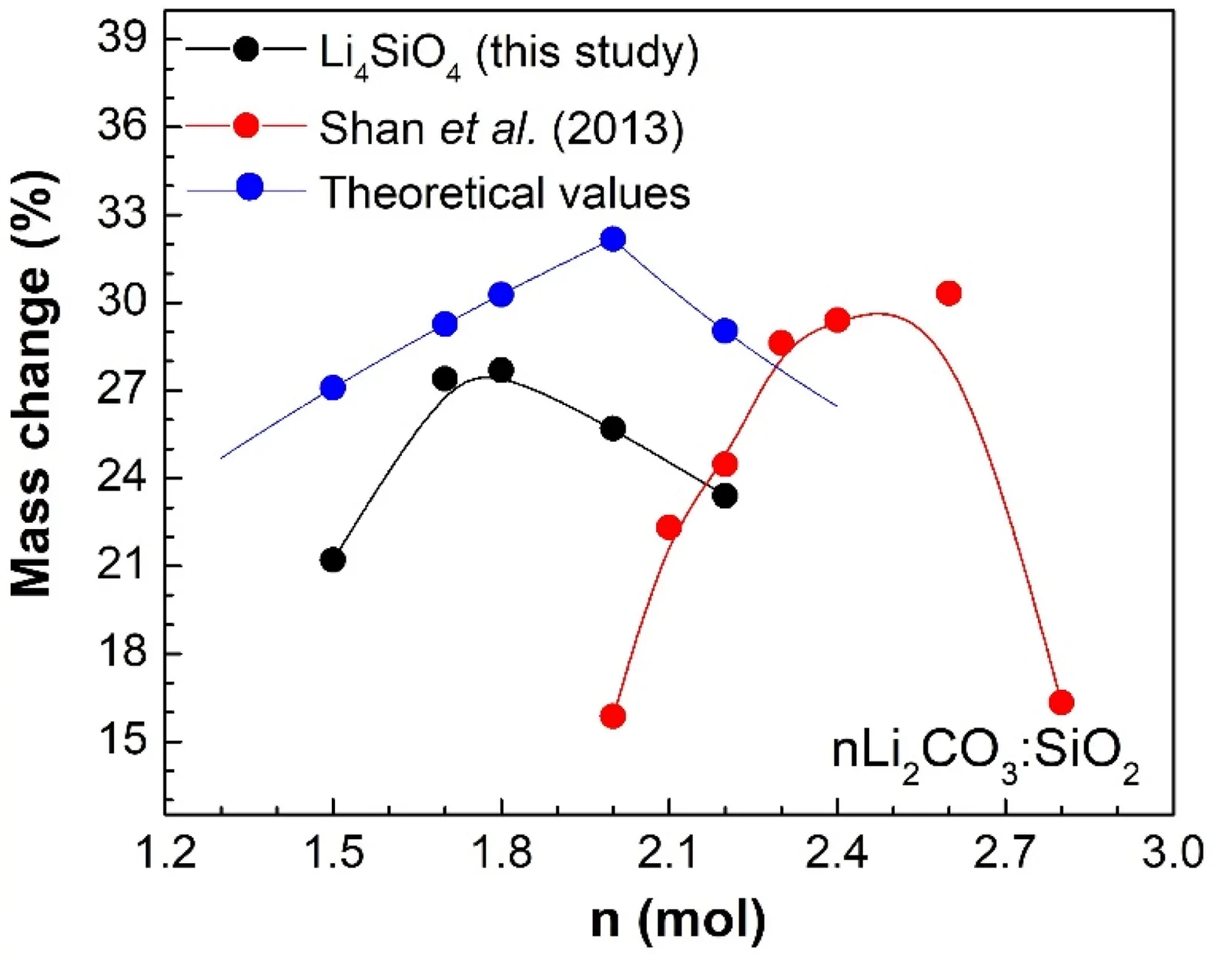
CO_2_ capture capacity at 650 °C under pure CO_2_ atmosphere as a function of the initial *n*Li_2_CO_3_–SiO_2_ molar ratio. Data points represent the synthesized Li_4_SiO_4_-based sorbents (black), the reference sample from Shan *et al.*^24^ (red), and the theoretical values calculated as described in Section 2.3 (blue).

For comparison, Fig. 3 also shows the theoretical CO_2_ capture capacity for each composition, calculated according to reaction (2), along with the experimental values reported by Shan *et al.*^24^ for samples prepared from a commercial diatomite source (Shanghai Fengxian Reagent Co. Ltd, China; 75 wt% SiO_2_). For *R* > 1.8, a pronounced drop in experimental CO_2_ capture capacity is observed. Notably, the experimental curve for *R* > 1.8 exhibits a slope parallel to the theoretical trend, suggesting that this decline is directly governed by the proportion of the active Li_4_SiO_4_ phase present. Mechanistically, this decay can be attributed to the presence of unreacted Li_2_CO_3_ (as confirmed by XRD in Fig. 2), which acts as an inert mass, which potentially alters diffusion pathways during carbonation.^24^ Remarkably, a maximum in capture capacity is observed across all profiles. However, the exact position of this maximum for natural Si sources is strongly dictated by the specific physicochemical characteristics of the diatomite. In particular, the structural nature of the silicon-bearing phases directly modifies the solid-state reactivity during synthesis, shifting the optimal composition.

Finally, complete carbonation of the Li_4_SiO_4_-based sorbents obtained from ball milled mixtures of *n*Li_2_CO_3_–SiO_2_ with various molar ratios was verified by XRD (Fig. S3). The phases corresponding to the products of carbonation (eqn (1)), specifically Li_2_CO_3_ and Li_2_SiO_3_ (ICDD 00-029-0828), were identified alongside remnants of unreacted Li_4_SiO_4_. In the next sections, the performance of the Li_4_SiO_4_ synthesized from 1.7Li_2_CO_3_–SiO_2_ milled mixture from natural diatomite (LD) was contrasted against a benchmark sorbent prepared from a commercial SiO_2_ source at a stoichiometric 2 : 1 molar ratio (LS).

### 3.2. Pressure-composition isotherms and thermodynamic analysis

To determine the thermodynamic parameters of the Li_4_SiO_4_–CO_2_ reaction system, pressure-composition isotherms (PCIs) were measured at temperatures between 575 °C and 675 °C (Fig. 4A). The primary aim was to establish whether utilizing diatomite as a precursor for Li_4_SiO_4_ (LD) influences the experimental equilibrium data compared to a reference material synthesized from commercial reagents (LS800). Specifically, the dependence of the equilibrium CO_2_ partial pressure (*P*_CO_2__) on temperature is critical for evaluating the potential application of these sorbents in industrial processes.

**Fig. 4:**
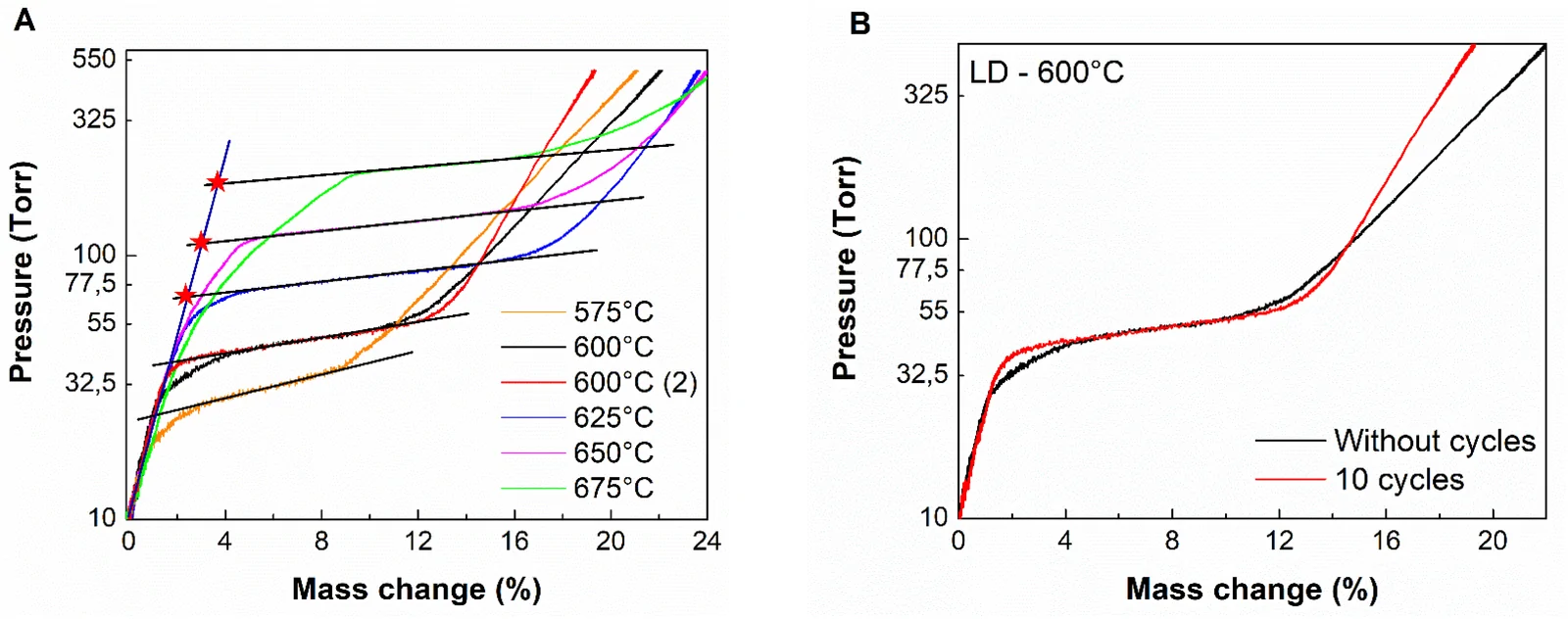
Pressure-composition isotherms for LD carbonation at different temperatures (A) and comparison of two isotherms obtained at 600 °C corresponding to the cycle 1 and cycle 10 (B).

As a typical behavior, all PCI curves display a single plateau at each temperature (Fig. 4A). Each of them exhibited a slight slope under isothermal conditions, an effect more pronounced at lower temperatures (575 °C and 600 °C) due to kinetic limitations (slow carbonation rates). This is further supported by comparing the PCI curves obtained at 600 °C for the 1st and 10th cycles (Fig. 4B), which demonstrate that LD approaches equilibrium more effectively after undergoing previous CO_2_ capture-desorption cycles. A similar trend was observed for the LS reference material (Fig. S4), where a slower pressure ramp was used to partially mitigate the effects of slow kinetics.

Assuming unit activities for the solid phases and ideal gas behavior for CO_2_, the temperature dependence of the equilibrium partial pressure (*P*_eq_) for reaction (1) can be expressed directly by the van 't Hoff equation:4
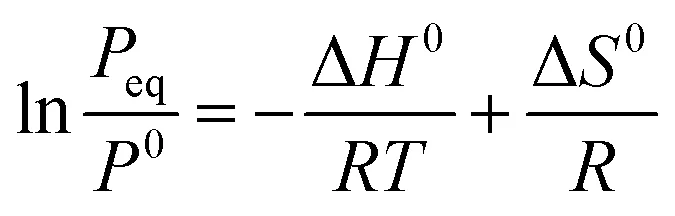
where *P*^0^ = 1 bar, Δ*H*° and Δ*S*° are the standard enthalpy and entropy of reaction, respectively, *R* is the universal gas constant, and *T* is the absolute temperature.

To determine the equilibrium pressures from each isotherm, a graphical criterion was applied. Independent linear fits were performed on the initial section (low CO_2_ capture capacity) and the plateau region of each PCI curve. The intersection of these two lines was considered as the equilibrium pressure (*P*_eq_) at the corresponding temperature, as indicated by the red stars in Fig. 4 and S4. These values (Table S1) were subsequently used to construct the van't Hoff graphs by plotting the logarithm of the equilibrium CO_2_ pressures *versus* the inverse of the temperature (Fig. 5), according to eqn (4). For the LS800 sorbent, the pressure was increased at a lower rate of 0.005 torr s^−1^ to improve the approximation to equilibrium. Furthermore, as previously discussed, at lower temperatures, the slower kinetics prevented the system from reaching equilibrium within the same timeframe (Fig. 5B). This is reflected in the van 't Hoff plot by the deviation from linearity of the point corresponding to the lowest temperature. The different equilibration rates between the LD and LS800 samples are also evidenced by their measured temperature ranges, which allowed equilibrium pressures to be obtained at lower temperatures for LD (Fig. S5). The Δ*H*° values determined from the slopes of these linear fits (Fig. 5), were 135 ± 4 kJ mol^−1^ for LD and 139 ± 5 kJ mol^−1^ for LS800. The close agreement between these values indicates that both sorbents undergo the same carbonation/decarbonation reaction, suggesting that the differences observed in their PCI behavior are mainly associated with kinetic and microstructural effects rather than changes in the intrinsic reaction thermodynamics.

**Fig. 5:**
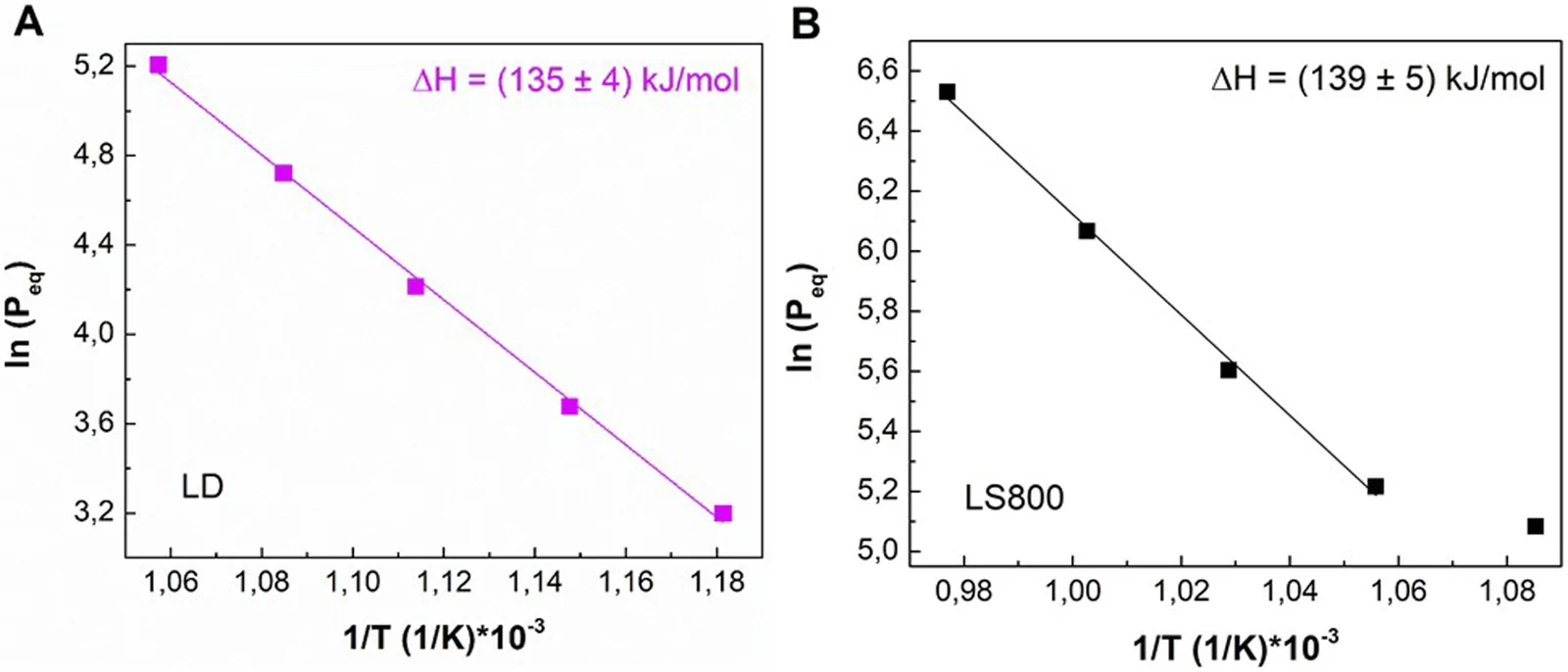
van't Hoff graphs for LD (A) and LS800 (B) derived from equilibrium CO_2_ pressures at different temperatures.

These experimental enthalpies fall well within the range established by comparable studies in the literature for Li_4_SiO_4_ (118–165 kJ mol^−1^).^31–33^ Specifically, Yang *et al.*^32^ proposed a theoretical value of Δ*H* ≈ 118 kJ mol^−1^ alongside with an experimental value of 162.5 kJ mol^−1^. Regarding the influence of materials composition, Chen *et al.*^33^ evaluated the effect of both Si precursors and metal dopants on the enthalpy of reaction. They reported that experimental values for metal-doped sorbents (145–165 kJ mol^−1^) were higher than those synthesized from different pure Si sources, observing that Δ*H* gradually increases with higher dopant content. Considering these reported trends for both pure and doped Li_4_SiO_4_, our results indicate that the natural impurities present in the diatomite (LD) do not exert a noticeable effect on the thermodynamic stability of Li_4_SiO_4_ when compared to the sorbent prepared from commercial SiO_2_ (LS800).

### 3.3. Sorption kinetic properties of Li_4_SiO_4_ synthesized from diatomite

Dynamic carbonation/decarbonation of the LD sorbent was evaluated *via* simultaneous TG-DSC to compare its performance under different CO_2_ partial pressures: pure CO_2_ (acting as the upper benchmark for uptake capacity) and 10% CO_2_ (representative of post-combustion flue gas conditions). The resulting mass change profiles and DSC thermograms, obtained during heating from room temperature (r.t.) to 800 °C, are displayed in Fig. 6A and B, respectively.

**Fig. 6:**
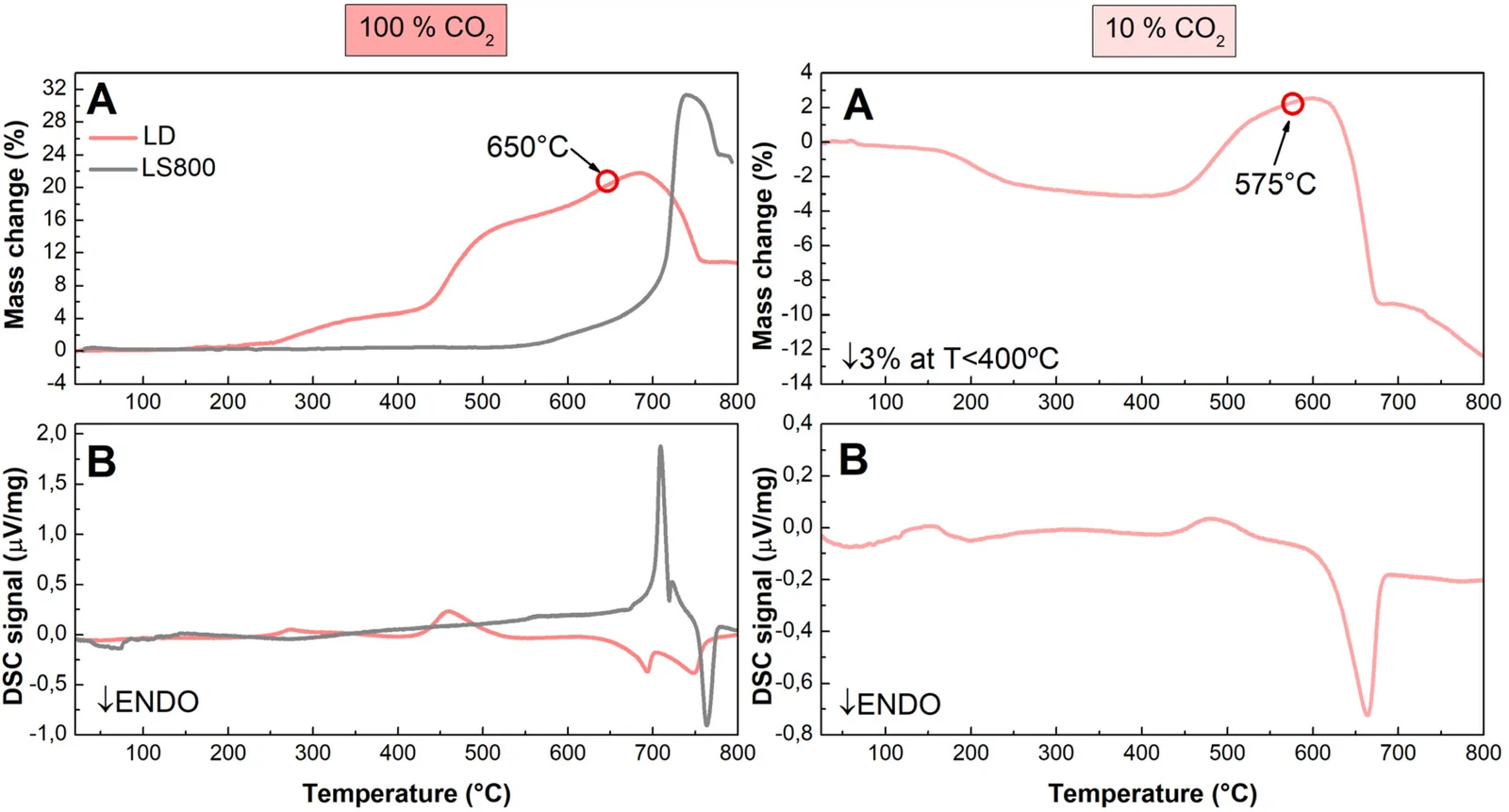
Combined thermal analysis TG (A) and DSC (B) of the LD in pure CO_2_ flow (left side) and 10% CO_2_ (right side). Curve corresponding with LS800 as reference is also included.

Under pure CO_2_, the TG curve (Fig. 6A) shows an initial mass gain from r.t. up to 430 °C due to superficial CO_2_ chemisorption. This is followed by a sharp mass increase driven by bulk Li_4_SiO_4_ carbonation, reaching a maximum CO_2_ uptake of ∼21 wt% at 680 °C. This dynamic correlates well with the DSC profile (Fig. 6B), which exhibits two distinct exothermic peaks centered at 270 °C and 470 °C, corresponding to the surface and bulk carbonation processes of LD, respectively. Interestingly, when comparing this behavior with the LS800 (Fig. 6A) and LS600 (Fig. S6A), both the onset temperature of carbonation and the maximum CO_2_ uptake for both LS shift to a higher temperature (740 °C). This indicates a significantly enhanced reactivity toward CO_2_ for the sorbent synthesized from natural diatomite, which effectively lowers the thermal requirements of the capture process compared to the benchmark material.

In contrast, under a 10% CO_2_ atmosphere, both the carbonation rate and the total CO_2_ uptake are lower under dynamic conditions, reaching only 6 wt%. During the initial heating stage in 10% CO_2_, a slight mass loss dominates due to the desorption of pre-adsorbed surface species, followed by the main CO_2_ capture event that peaks at 600 °C. This capture process is also mirrored by an exothermic event in the DSC curve.

Following the carbonation peaks, a decarbonation step occurs under both conditions, shifting toward a lower temperature in the 10% CO_2_ environment. The endothermic phenomena identified in the DSC curves show an excellent correlation with these mass loss steps. Under pure CO_2_, two endothermic events are resolved: the first at 692 °C is associated with the melting of the Li_2_CO_3_, while the second at 748 °C corresponds to the decarbonation reaction. Conversely, only a single endothermic decarbonation peak is detected for the 10% CO_2_ condition, with a superposition of both endothermic events.

To further understand these non-isothermal TGA-DSC trends, the microstructural evolution, phase purity, and sorption stability of LD were systematically evaluated against LS600 and LS800 (Table S2 and Fig. S6–S8). Under identical synthesis conditions (600 °C, 24 h), LD achieves complete Li_4_SiO_4_ phase purity with a refined crystallite size of 60 nm (Fig. 2 and Table S2). In contrast, the commercial control sample LS600 prevents complete solid-state reaction, retaining unreacted Li_2_CO_3_ and exhibiting a larger crystallite size of 83 nm. The commercial precursor requires a higher treatment temperature of 800 °C (LS800) to reach complete phase transformation, which significantly increases the crystallite size to 103 nm (Fig. S6B and Table S2).

Interestingly, all final calcined products display similarly low specific surface areas (∼1 m^2^ g^−1^, Table S2). Remarkably, LD achieves full phase formation at 600 °C despite raw diatomite having a lower initial surface area (45 m^2^ g^−1^) than commercial SiO_2_ (660 m^2^ g^−1^). This proves that natural impurities/additives in the diatomite matrix effectively lower the thermal activation energy for Li_4_SiO_4_ formation, an effect that dominates over simple initial precursor surface area or thermal sintering. Consequently, non-isothermal profiles confirm that both commercial references (LS600 and LS800) require higher temperatures to reach maximum CO_2_ capture rates compared to LD.

Based on previous dynamic TG-DSC findings (Fig. 6), 650 °C and 575 °C were selected as the optimal working temperatures for subsequent studies under pure CO_2_ and 10% CO_2_, respectively. These values are strategically located just below the maximum mass gain temperature, ensuring peak capture efficiency immediately before the carbonation reaction begins to reverse.

The cyclic stability and reversibility of the LD sorbent was evaluated over 10 carbonation/regeneration cycles under both pure and 10% CO_2_ atmospheres (Fig. 7). Each experiment began with an initial preheating stage under an inert atmosphere to condition the sorbent by removing pre-adsorbed surface species. Both the carbonation and decarbonation stages were considered complete once the mass change rate fell below a predefined threshold (see Section 2.3), indicating that the reaction was approaching completion. As shown in Fig. 7, the LD sorbent exhibits excellent reversibility under both concentrated and diluted CO_2_ conditions. On the contrary, LS600 suffers severe capacity loss upon cycling due to incomplete reaction (Fig. S8). Notably, the carbonation stages of LD under 10% CO_2_ (575 °C) required approximately twice the time to reach completion compared to pure CO_2_ (650 °C). This behavior is mainly governed by kinetic limitations at the lower operating temperature (575 °C), alongside a reduced CO_2_ partial pressure. Although the equilibrium pressure also drops significantly at this lower temperature (from *ca.* 120 to 25 torr), preserving a thermodynamic driving force, the decreased thermal energy and lower reactant concentration overall slowdown the reaction rate. Regarding the performance, while the theoretical CO_2_ capture capacity calculated using eqn (3) is 29 wt%, the experimental values achieved in this work reached a remarkable 27 wt% under pure CO_2_ and 22 wt% under 10% CO_2_ (93% and 76% of theoretical capacity, respectively).

**Fig. 7:**
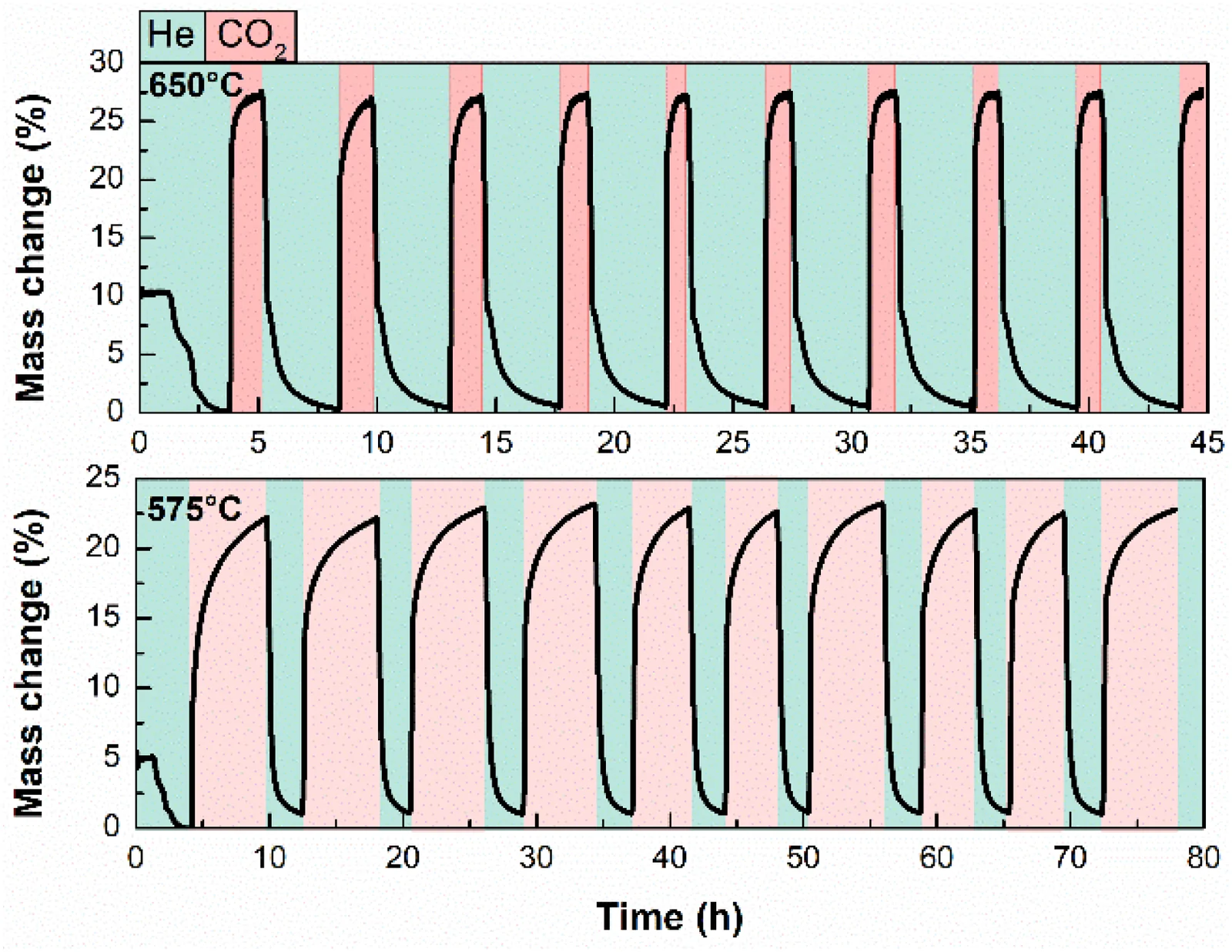
Cyclic CO_2_ capture/release performance of the LD sorbent over 10 cycles under: pure CO_2_/inert atmosphere (at 650 °C) and a 10% CO_2_/inert atmosphere (at 575 °C).

These values demonstrate a highly efficient conversion of the Li_4_SiO_4_ active phase into carbonated products. Furthermore, this capture capacity remained practically constant throughout the successive cycles, confirming the outstanding cyclic stability of the material under the investigated experimental conditions.

To evaluate the structural and microstructural changes induced by the carbonation and decarbonation reactions, the samples were analyzed by XRD (Fig. 8) and SEM (Fig. 9) before and after the 10th cycle. The diffraction patterns confirmed the formation of the expected carbonation products according to eqn (1), namely Li_2_CO_3_ and Li_2_SiO_3_, alongside a remnant fraction of unreacted Li_4_SiO_4_. Interestingly, the secondary phase LiFeO_2_, originating from the natural impurities of the diatomite, remained stable after cycling.^34^ Furthermore, the presence of Li_2_CaSiO_4_ was identified as a product of carbonation at 575 °C under a 10% CO_2_. While the stability of LiFeO_2_, suggests it does not negatively affect the material's structural durability and could instead contribute to its high sintering resistance, the Li_2_CaSiO_4_ phase might actively participate in the interaction with CO_2_.^35,36^ The detection of these Fe- and Ca-bearing phases is fully consistent with the elemental composition of the starting diatomite.

**Fig. 8:**
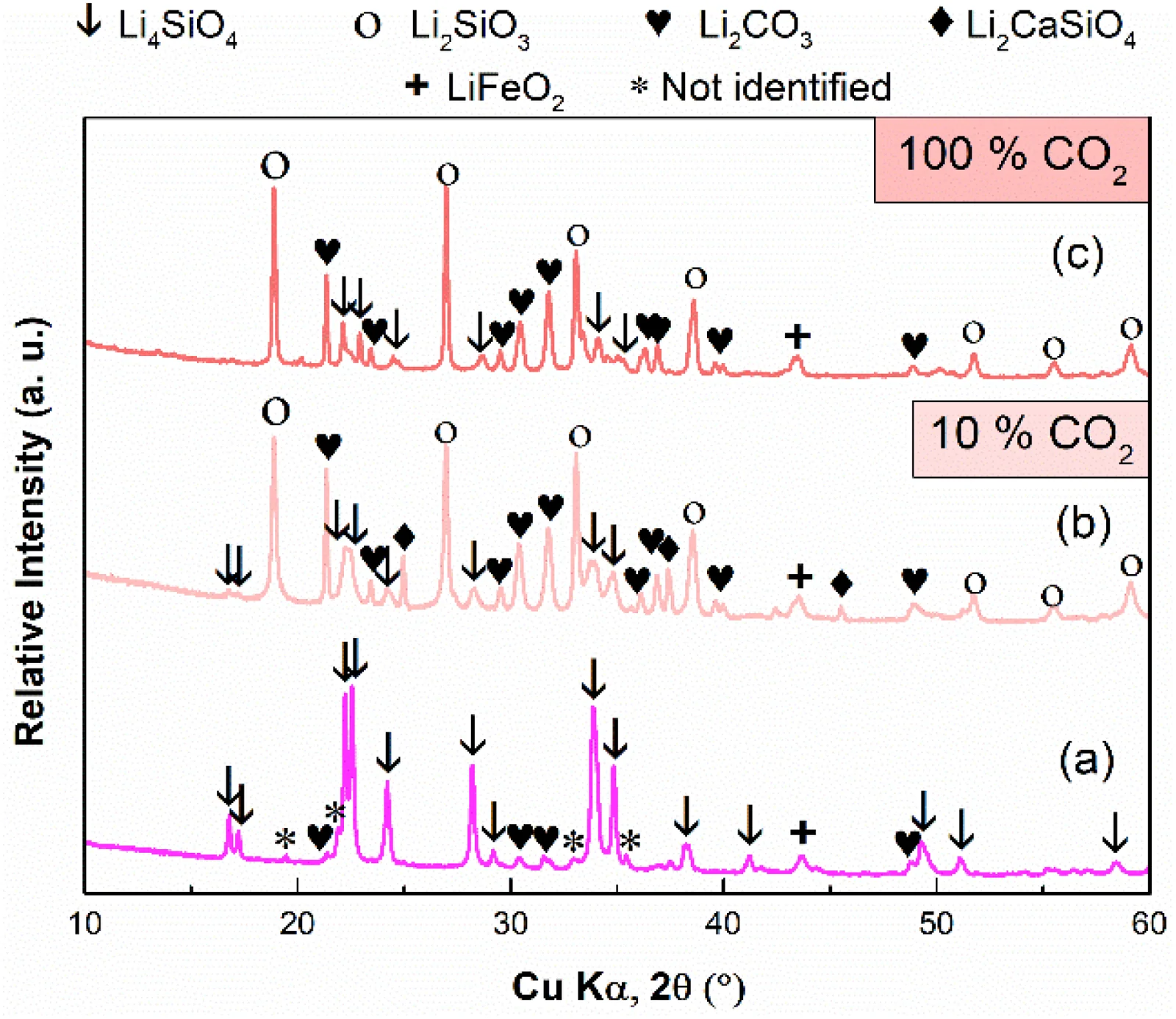
XRD patterns of LD after synthesis (a) and over 10 cycles of CO_2_ capture/release under: a 10% CO_2_/inert atmosphere at 575 °C (b) and pure CO_2_/inert atmosphere at 650 °C (c).

**Fig. 9:**
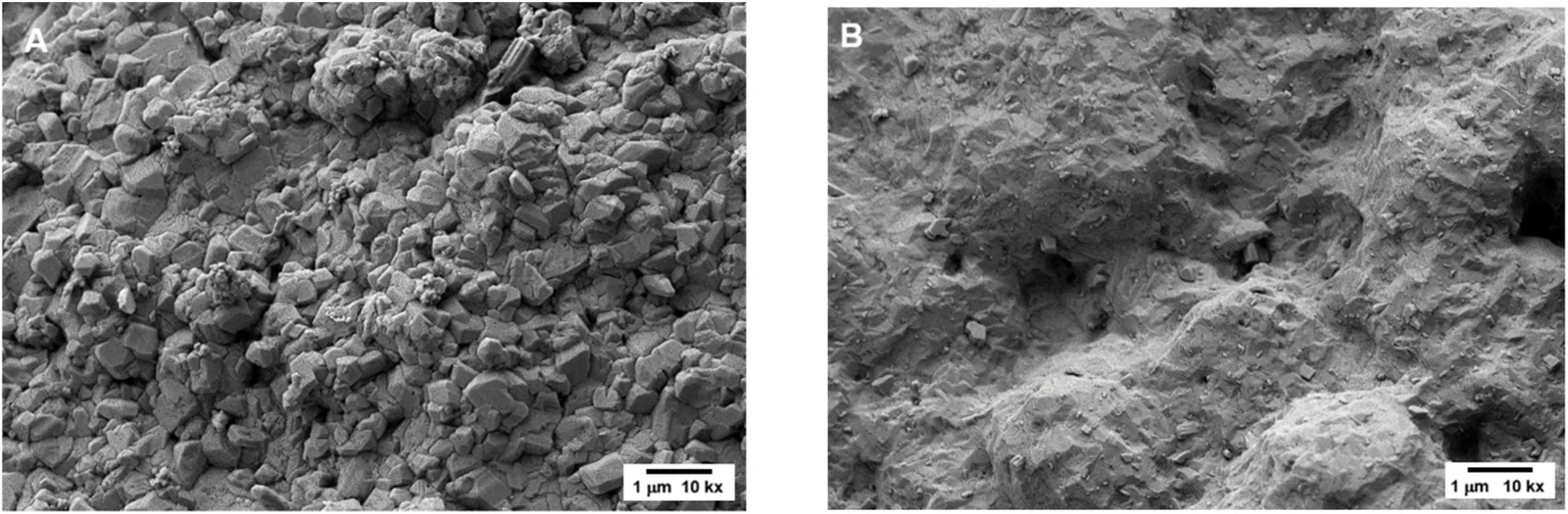
SEM micrographs of (A) as-synthesized Li_4_SiO_4_ from diatomite and (B) after 10 cycles of carbonation/decarbonation under 10% CO_2_ at 575 °C.

A detailed inspection of the main diffraction peaks of LD compared to LS800 reveals a shift toward higher 2*θ* angles specifically for the (001) plane (Fig. S9), whereas the positions of other diffraction peaks remain practically unchanged. This anisotropic behavior could tentatively be attributed to the partial substitution of Li^+^ by Mg^2+^ in the crystal structure.^37^ Given the smaller ionic radius of Mg^2+^ relative to Li^+^, its incorporation may induce a preferential contraction along the *c*-axis with minimal effect on the *a* and *b* lattice parameters. Notably, this selective lattice contraction is strictly observed in LD and absent in both LS800 and LS600 (Fig. S6), confirming that this structural modification is a direct benefit derived from the natural mineral matrix.

Conversely, other potential substitutions involving cations present in the diatomite (see Table 1), such as K^+^, Na^+^ or Ca^2+^, would induce a lattice expansion, resulting in a shift toward lower 2*θ* values. Similarly, alternative substitutions in Li_4_SiO_4_ involving the replacement of Si^4+^ by Al^3+^ have been reported to cause lattice expansion (a leftward shift), since Al^3+^ (0.039 nm) possesses a larger ionic radius than Si^4+^ (0.026 nm).^38^ Because the experimental results suggest a net lattice contraction when using diatomite as the silicon source, the observed shift can reasonably be associated with the potential substitution of Li^+^ by Mg^2+^.

The morphological features of Li_4_SiO_4_ before and after CO_2_ sorption cycles under 10% of CO_2_ were examined by SEM (Fig. 9). The as-synthesized LD sample was composed of faceted particles ranging from 0.2 to 0.6 µm in size, forming a slightly intertwined grain framework (Fig. 9A). These particles are smaller than those observed in LS600 (rounded, >1 µm) and LS800 (several µm) (Fig. S7). After 10 sorption cycles, the surface of some particles appear covered by a molten layer. This liquid phase can be attributed to the formation of a eutectic mixture between Li_2_CO_3_ and K and/or Na carbonates.

To better understand the distribution of these phases, elemental mapping of the carbonated LD sample was performed (Fig. S10). Unlike most elements originating from the diatomite impurities (such as Mg, Na, Ca, and Fe), which are uniformly distributed across the surface, Al segregates into well-defined, isolated zones. The potassium (K) is also widely dispersed, although it exhibits some zones with higher localized concentrations. This widespread presence of Na and K closely correlates with the extensive molten layer observed, tracking the formation of binary Li–Na and/or Li–K or ternary Li–Na–K eutectic carbonate mixtures.

The occurrence of a liquid phase during LD carbonation is further supported by DSC studies (Fig. S11A), where two exothermic peaks associated with solidification are systematically observed across all *n*Li_2_CO_3_–SiO_2_ molar ratios when using diatomite as the silicon source. In contrast, the LS800 sample exhibits no exothermic events during cooling (Fig. S11B). Previous studies^39–41^ have reported that such molten phases promote CO_2_ diffusion through the solid product layer. Consequently, the LD sorbent exhibited enhanced CO_2_ capture capacity and kinetics compared to LS800, driven by the reaction of K- or Na-based additives with the Li_4_SiO_4_ shell formed during carbonation.

The evaluation of the activation energy (*E*_a_) provides crucial insights into the energy barrier and reactivity of the LD sorbent during CO_2_ capture. For this purpose, the Kissinger method was applied to the dynamic thermochemical data.^30^ This differential method is widely preferred as it enables the determination of *E*_a_ directly from the maximum reaction rates at varying heating rates, without requiring prior knowledge or assumptions about the reaction mechanism. The linear formulation of the Kissinger method is expressed as follows:5
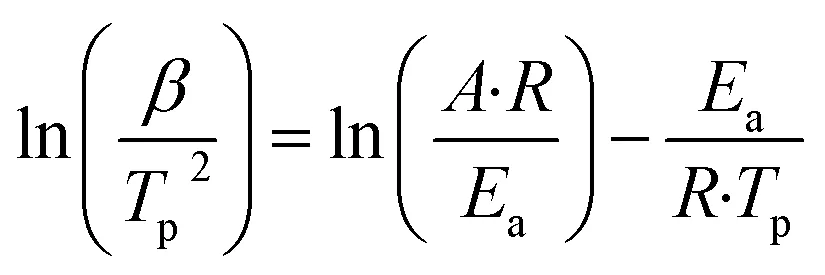
where *β* is the heating rate (°C min^−1^), *T*_p_ is the absolute peak temperature (K) corresponding to the maximum carbonation rate, *A* is the pre-exponential factor (min^−1^) and *R* is the universal gas constant (8.314 J (mol^−1^ K)). Consequently, a plot of ln(*β*/*T*_p_^2^) *versus* 1/*T*_p_ yields a straight line, from whose slope (−*E*_a_/*R*) the activation energy of the carbonation process can be straightforwardly calculated.

To obtain the required experimental *T*_p_ values for the carbonation process, approximately 27 mg of the LD sorbent was conditioned before each run by heating to 650 °C under inert atmosphere to remove pre-adsorbed species. The sample was then cooled to 200 °C, and the CO_2_ pressure was adjusted to 3000 torr. This pressure was strategically selected to ensure that the forward carbonation reaction was thermodynamically favored over the reverse decarbonation reaction, even at high temperatures (as supported by Fig. 4). Once the pressure stabilized, non-isothermal carbonation runs were performed at different heating rates, recording the mass evolution over time. To reduce experimental noise, the raw mass change profiles were smoothed using an adjacent-point averaging algorithm (Fig. 10A). The curves of derivative of the relative mass change with respect to temperature were subsequently calculated *via* finite differences, followed by a second adjacent-point smoothing step to precisely identify the carbonation *T*_p_ values (Fig. 10B). The peak temperatures (*T*_p_) were then determined from the maxima of the first derivative of the relative mass change with respect to temperature (Fig. 10B).

**Fig. 10:**
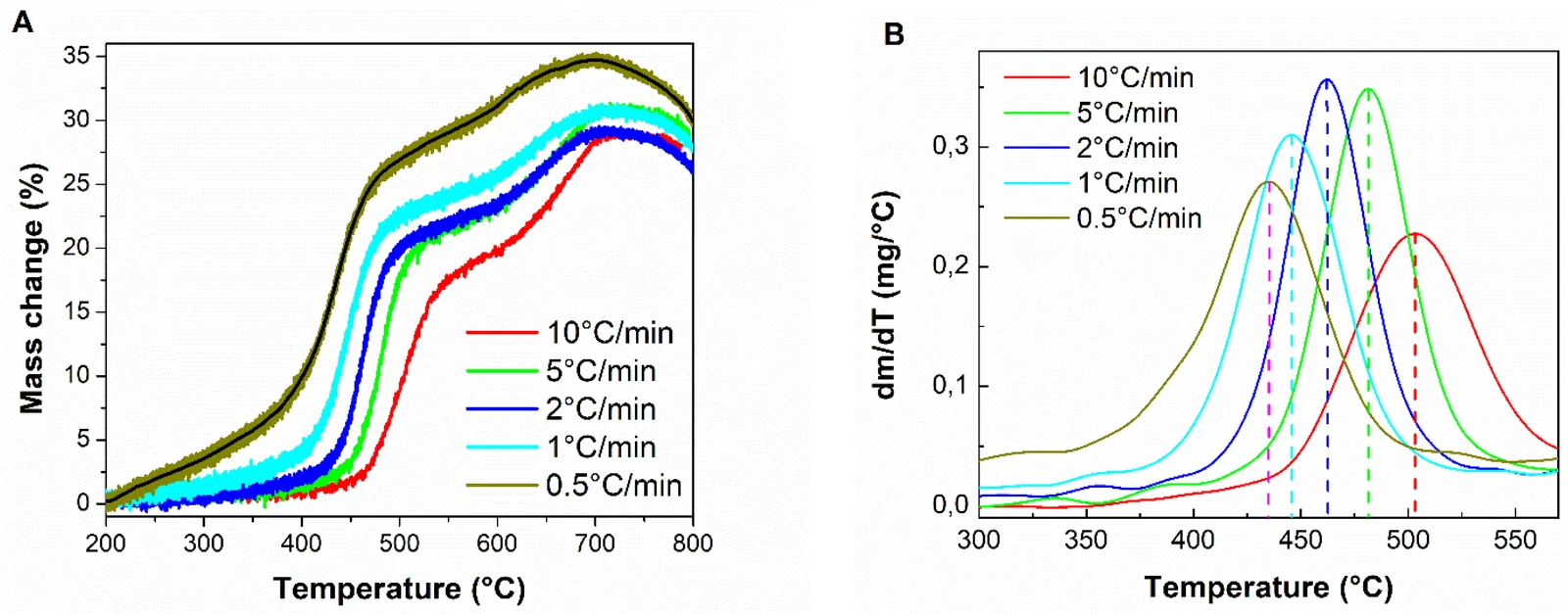
Carbonation of LD carried out in TG at different heating ramp. (A) CO_2_ capture capacity (%) as a function of temperature. (B) Derivative of mass change respect temperature as a function of temperature.

A similar procedure was employed to investigate the decarbonation kinetics. First, 150 mg of a carbonated sample was prepared by fully carbonating the conditioned LD sorbent under a CO_2_ atmosphere until saturation. Non-isothermal decarbonation tests were then conducted using this fully carbonated sample under an Ar flow (100 sccm) at different heating rates. The high gas flow rate, together with the low sample mass, was selected to ensure efficient removal of the released CO_2_ from the vicinity of the sample, thereby minimizing the influence of the gas atmosphere on the decarbonation process. To reduce experimental noise, the raw mass change profiles were smoothed using the same procedure previously described for carbonation curves (Fig. 11A). The curves of derivative of the relative mass change with respect to temperature were subsequently calculated to identify the decarbonation *T*_p_ values (Fig. 11B).

**Fig. 11:**
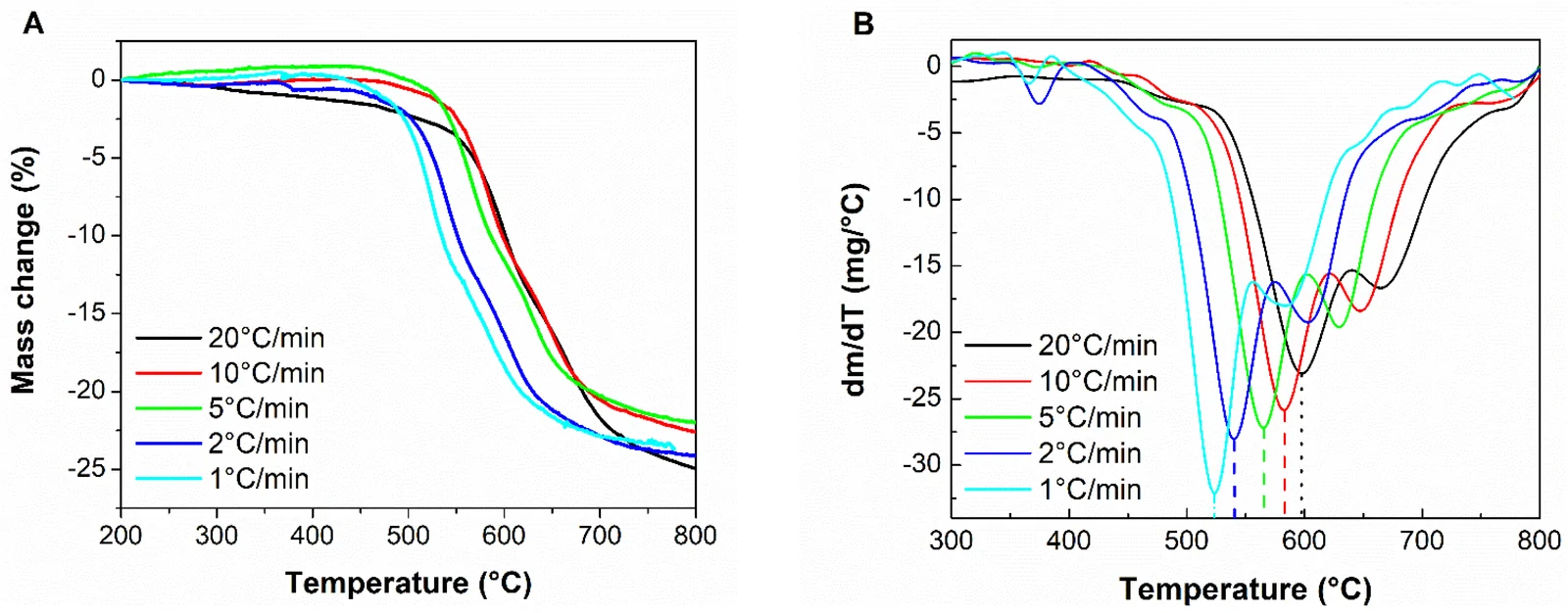
Decarbonation of LD carried out in TG at different heating ramp. (A) CO_2_ capture capacity (%) as a function of temperature. (B) Derivative of mass change respect temperature as a function of temperature.

Using the experimental *T*_p_ values obtained at different heating rates for both carbonation and decarbonation, the Kissinger plots were constructed (Fig. 12), and the same experimental and mathematical treatments were applied to the LS reference for comparison. For the LD sorbent, the activation energy for decarbonation was determined to be 210 ± 4 kJ mol^−1^, which stands in excellent agreement with the values reported in the literature (around 230–350 kJ mol^−1^)^42,43^ Regarding carbonation, LD exhibited an *E*_a_ of 187 ± 7 kJ mol^−1^, falling well within the typically reported intervals of 90–300 kJ mol^−1^.^42–45^ This close agreement with previous literature validates both our experimental treatment and the applied kinetic analysis.

**Fig. 12:**
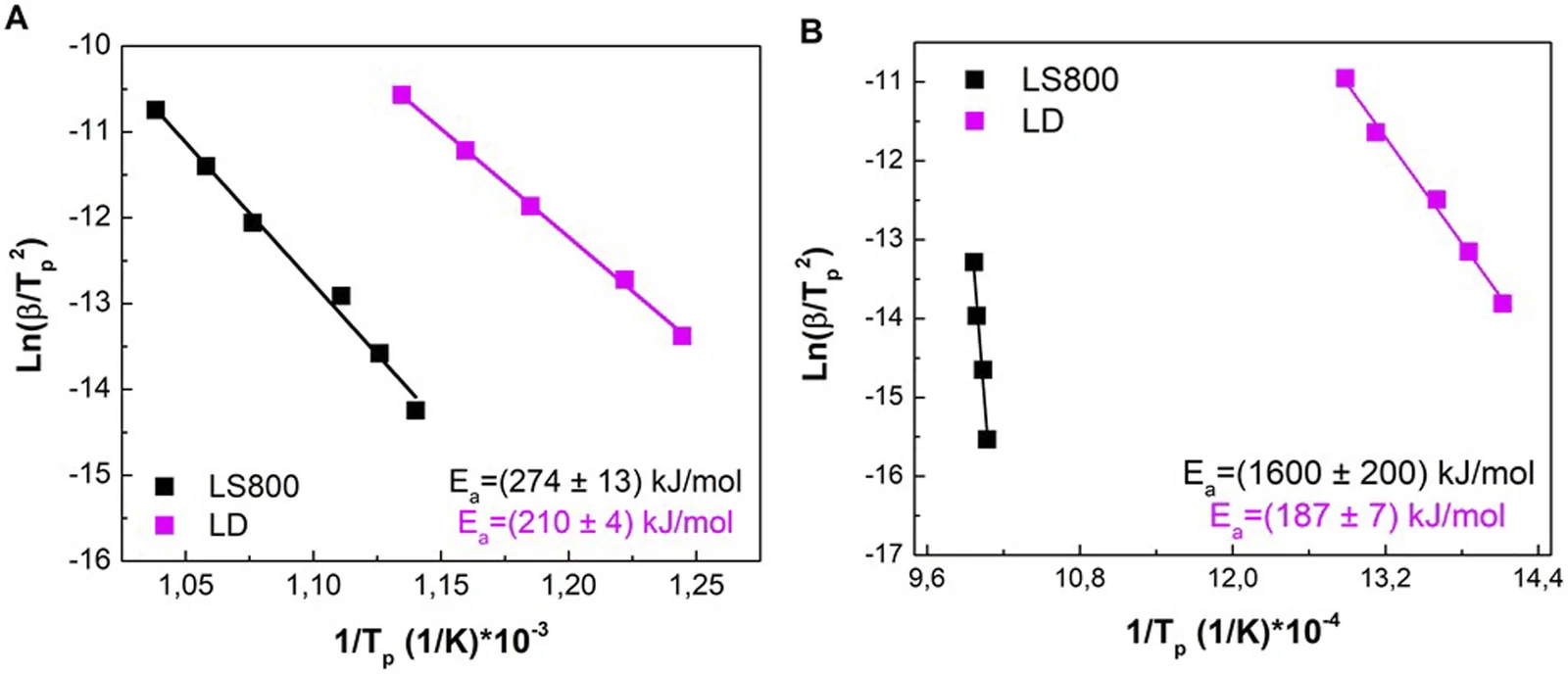
Activation energies determined by the Kissinger method for the decarbonation (A) and carbonation (B) of LD and LS800.

Conversely, a highly anomalous behavior was observed for the LS reference during carbonation, preventing a direct kinetic comparison for this specific process. For this material, the *T*_p_ values shifted into the range of 717–723 °C, which lies in the immediate vicinity of the Li_2_CO_3_ melting point. Within this specific temperature window, the relative mass change evolution and its corresponding derivative are heavily distorted by Li_2_CO_3_ melting. Under these overlapping conditions, the fundamental assumption of the Kissinger method, which requires a single, well-defined underlying kinetic process, is violated. In particular, the melting of Li_2_CO_3_ is a highly endothermic phase transition with a large latent heat of fusion. This severe endothermic heat effect directly opposes the exothermic nature of the carbonation reaction, significantly distorting the thermal response and shifting the peak temperatures. Consequently, the value obtained for LS800 carbonation cannot be interpreted as a true activation energy, but rather as an apparent energy heavily influenced by multiple concurrent physical and chemical phenomena.

Nevertheless, a robust comparison remains valid for the decarbonation process, where both materials were evaluated under identical conditions. Remarkably, the diatomite-derived Li_4_SiO_4_ (LD) displayed a lower decarbonation activation energy than the benchmark commercial reagent (LS800). This key finding reinforces the hypothesis that native impurities and/or the *in situ* formed secondary phases within the natural diatomite matrix play a crucial role in lowering the energy barriers, thereby accelerating the reaction kinetics.

When compared to previously reported Li_4_SiO_4_ sorbents derived from Chinese diatomite,^24,46^ the present mechano-thermally synthesized sorbent (LD) offers notable synthesis and operational advantages. Initially, Li_4_SiO_4_ was synthesized by solid-state reaction requiring a calcination temperature of 700 °C and a large excess of lithium (Li_2_CO_3_ : SiO_2_ = 2.6 : 1) to promote phase formation, achieving an initial absorption capacity of 34.14 wt% under 50% CO_2_ at 700 °C.^24^ In a subsequent study using the same diatomite source, Shan *et al.*^46^ synthesized Li_4_SiO_4_*via* a wet impregnation–precipitation route followed by calcination at 600–700 °C for 4 h. When evaluated under 50% CO_2_ at 700 °C, this Li_4_SiO_4_ wet-synthesized sorbent exhibited faster adsorption kinetics and superior cyclic stability compared to its solid-state counterpart. While a direct comparison is limited by the different evaluation conditions (50% CO_2_ at 700 °C *vs.* 10% CO_2_ at 575 °C), our two-step mechano-thermal route enables low synthesis temperatures (600 °C) and substantially reduces raw lithium consumption by optimizing the molar ratio to 1.7–1.8. Under realistic, highly diluted flue gas conditions (10% CO_2_ at 575 °C), the LD sorbent maintains a stable capacity of 22 wt% (reaching 27 wt% under pure CO_2_) with excellent cyclic retention across 10 carbonation–decarbonation cycles. This high performance under low CO_2_ partial pressure is attributed to the synergistic action of natural impurities present in Argentine diatomite, which accelerate diffusion kinetics and lower the activation energy for decarbonation (210 kJ mol^−1^) without altering the thermodynamic stability of the material.

Mechanistically, these natural impurities play a synergistic role in enhancing the CO_2_ capture performance of the LD sample compared to its pure LS counterpart. On one hand, the *in situ* formation of an alkali-metal eutectic molten phase bypasses the diffusion limitations typical of the solid product layer, significantly accelerating the reaction kinetics and lowering the activation energy required for the diffusion process. On the other hand, the lattice contraction likely associated with Mg^2+^ substitution, alongside the dispersing effect of the stable LiFeO_2_ secondary phase prevents severe sintering and crystal aggregation. Together with the active participation of Ca-bearing phases, these structural and morphological modifications grant the LD sorbent a remarkably higher CO_2_ capture capacity on the active part, faster kinetic response, and superior structural stability over cycling than the synthetic LS reference. Ultimately, these findings demonstrate that the direct utilization of raw diatomite, without any pre-conditioning or purification treatments, effectively turns native impurities into beneficial dopants.

Overall, these findings demonstrate that using low-cost natural silicon precursors, such as Argentine diatomite, halloysite,^25^ perlite,^26^ or other natural silica sources,^24,46^ represents a generalizable and sustainable strategy for high-performance CO_2_ sorbent development. Rather than being a limitation, the inherent metallic impurities (Al, Fe, Mg, Na, K) serve as effective built-in dopants. Depending on the chemical environment, these ions can modify the Li_4_SiO_4_ crystal structure, promote the formation of molten eutectic phases that accelerate sorption kinetics, or prevent thermal sintering over extended cycling. Consequently, tapping into the intrinsic impurity profiles of natural minerals offers a practical, scalable route to bypass expensive chemical doping steps in industrial CO_2_ capture applications.

Nevertheless, further optimization strategies regarding the sorbent's microstructure and texture remain to be explored. Future work will focus on tuning key processing parameters, such as milling energy, calcination time and temperature, or incorporating an activation step *via* organic acid treatments, to unlock the full potential of these diatomite-derived materials for high-temperature CO_2_ capture.

## 4 Conclusions

In this work, a sustainable and efficient Li_4_SiO_4_ sorbent (LD) for high-temperature CO_2_ capture was successfully synthesized *via* a two-step mechano-thermal method, utilizing raw, unpurified natural diatomite from Río Negro (Argentina) as the sole silicon precursor. The initial *n*Li_2_CO_3_–SiO_2_ molar ratio was optimized between 1.7 and 1.8, effectively maximizing the active phase yield.

Comprehensive thermodynamic and kinetic evaluations demonstrated that the diatomite-derived sorbent exhibits improved carbon capture performance, achieving a maximum experimental CO_2_ capture capacity of 27 wt% under pure CO_2_ (650 °C) and maintaining a remarkable 22 wt% under a realistic, diluted 10% CO_2_ atmosphere (575 °C). Furthermore, the LD sorbent displayed excellent cyclic and structural stability over 10 carbonation–decarbonation cycles, outperforming the conventional reference sorbent synthesized from commercial-grade reagents. Thermodynamic and kinetic studies *via* the van't Hoff and Kissinger analysis quantified a reaction enthalpy of 135 kJ mol^−1^ and a decarbonation activation energy of 210 kJ mol^−1^. These results demonstrate that while the impurities in the diatomite-derived Li_4_SiO_4_ do not alter its thermodynamic stability, they exert an effect on the sorption kinetics, lowering the energetic barrier for diffusion and enhancing reaction rates compared to the synthetic benchmark.

Structural and morphological analyses provided crucial insights into the underlying mechanisms. It was revealed that the trace elements and native impurities naturally present in the Argentine diatomite served as beneficial, built-in multi-functional dopants. Specifically, the native impurities appear to induce a beneficial lattice contraction, presumably *via* Mg^2+^ substitution, while promoting the *in situ* formation of a stable LiFeO_2_ secondary phase alongside an alkali-metal eutectic molten phase during high-temperature operation. This synergistic mechanism effectively bypassed solid-state diffusion limitations, accelerated carbonation kinetics.

Ultimately, these findings validate the direct utilization of raw regional diatomite without expensive pre-purification treatments, turning geographic availability into a strategic and competitive economic advantage. This study establishes a robust baseline for the development of advanced lithium-silicate sorbents tailored for practical carbon capture and storage (CCS) applications. Future work will address further microstructural optimizations, including the adjustment of milling energy and the exploration of organic acid activation steps, to fully unlock the potential of these -derived materials.

## Author contributions

E. D. T: conceptualization, data curation, formal analysis, methodology, investigation. M. L. G: conceptualization, methodology, investigation, validation, writing – review & editing. P. A. L: conceptualization, formal analysis, investigation, supervising, writing – review & editing. F. C. G: investigation, conceptualization, writing – original draft, writing – review & editing.

## Conflicts of interest

The authors declare that there are no conflicts of interest.

## Supplementary Material

RA-016-D6RA05886H-s001

## Data Availability

The data that support the findings of this study are included in this published article. Supplementary information (SI): Fig. S1–S11, Tables S1 and S2. See DOI: https://doi.org/10.1039/d6ra05886h.
