# Supplementary material for: Development of Li4SiO4 sorbents from Argentine diatomite for high-temperature CO2 capture
Source: RSC Adv. 2026 Sep 7;16(43):53208–21. doi: 10.1039/d6ra05886h (PMC13549009; doi:10.1039/d6ra05886h)
Supplement: Supplementary file 1 [file RA-016-D6RA05886H-s001.pdf]

Supplementary Information (SI) for RSC Advances.
This journal is © The Royal Society of Chemistry 2026

Development of Li_4SiO_4 sorbent from Argentine diatomite for high-temperature CO_2 capture

Eider David Torres[a], María Laura Grasso[b], Fabiana Gennari[a,b,c*] and Pierre Arneodo Larochette[a,b,c]

[a] National University of Cuyo-Balseiro Institute, Av. Bustillo 9500, R8402AGP S.C. de Bariloche, Río Negro, Argentina.

[b] Bariloche Atomic Center-National Atomic Energy Commission (CAB-CNEA), Av. Bustillo 9500, R8402AGP S.C. de Bariloche, Río Negro, Argentina.

[c] National Council for Scientific and Technical Research (CONICET), Av. Bustillo 9500, R8402AGP, S.C. de Bariloche, Río Negro, Argentina.

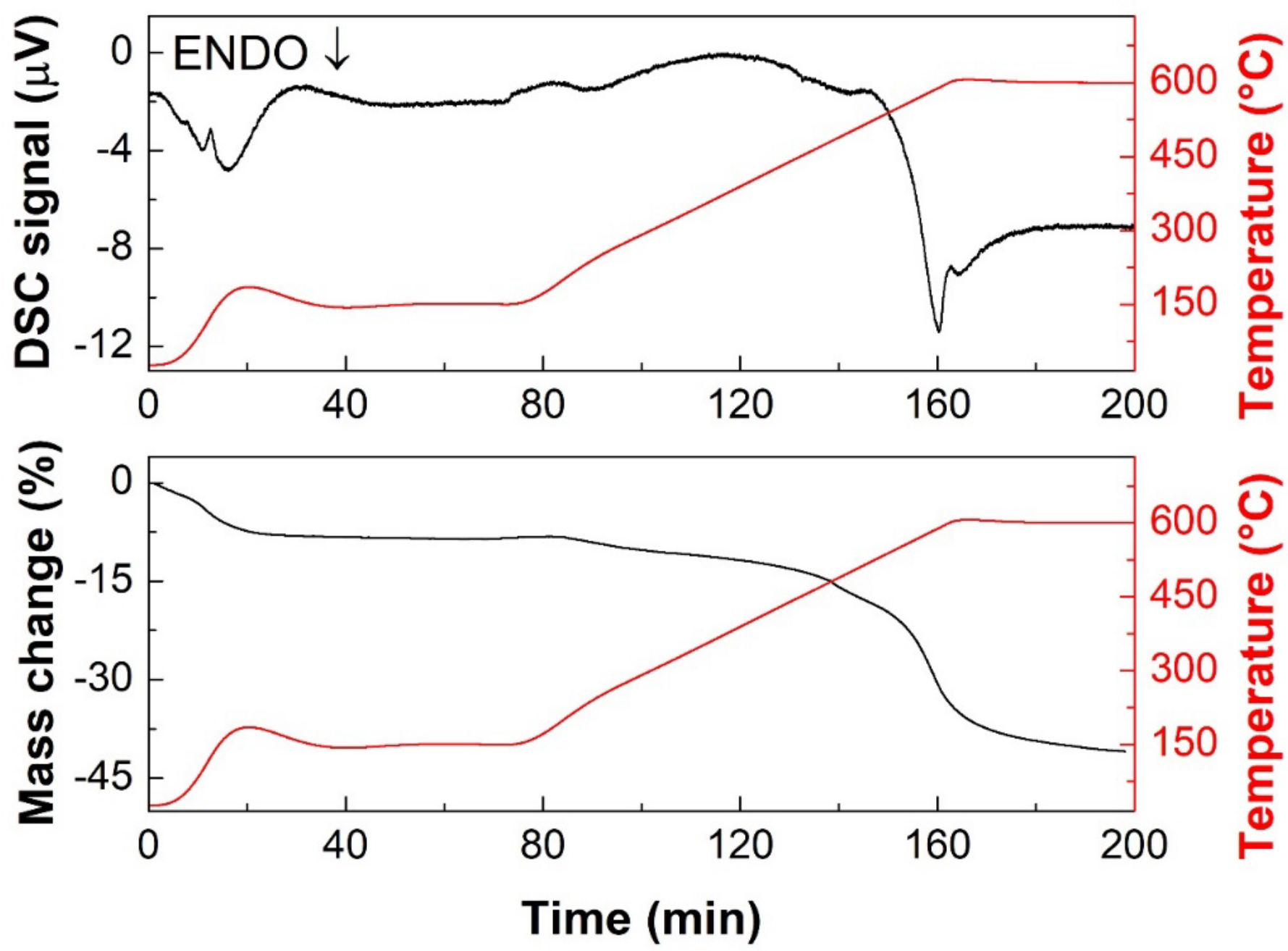

Figure S1. TG-DSC curves of the ball milled Li_2CO_3-SiO_2 mixture (1.7:1 molar ratio) heated in air (50 sccm) in two successive steps: (i) from room temperature to 150 °C, and (ii) from 150 to 600 °C at a heating rate of 5 °C/min, followed by a 2 h dwell time at 600 °C.

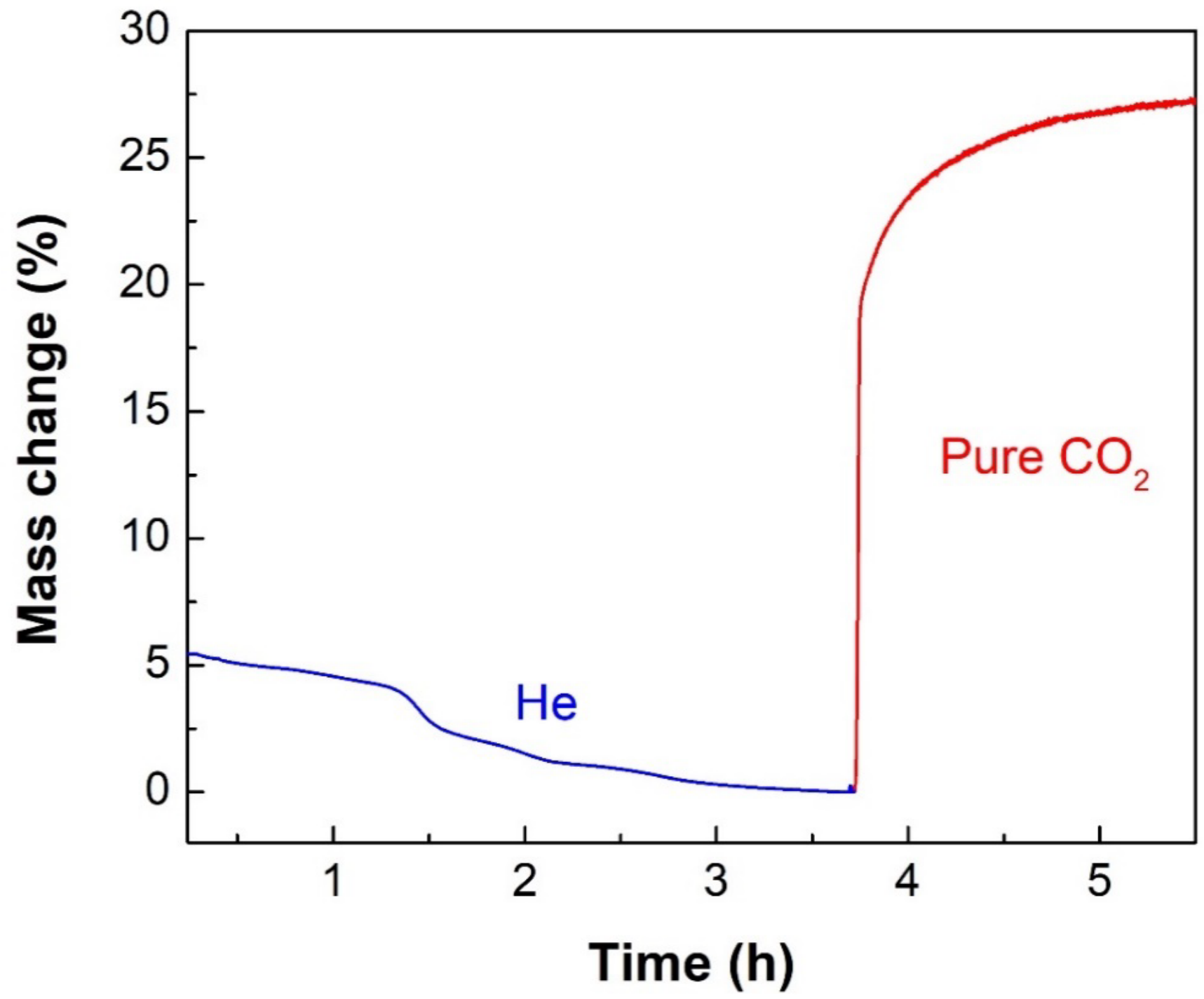

Figure S2. Thermogravimetric analysis of LD after carbonation in a flow of pure CO_2 (red) at 650 °C with preheating under inert gas (blue). The TG curve shows the relative mass change during heating.

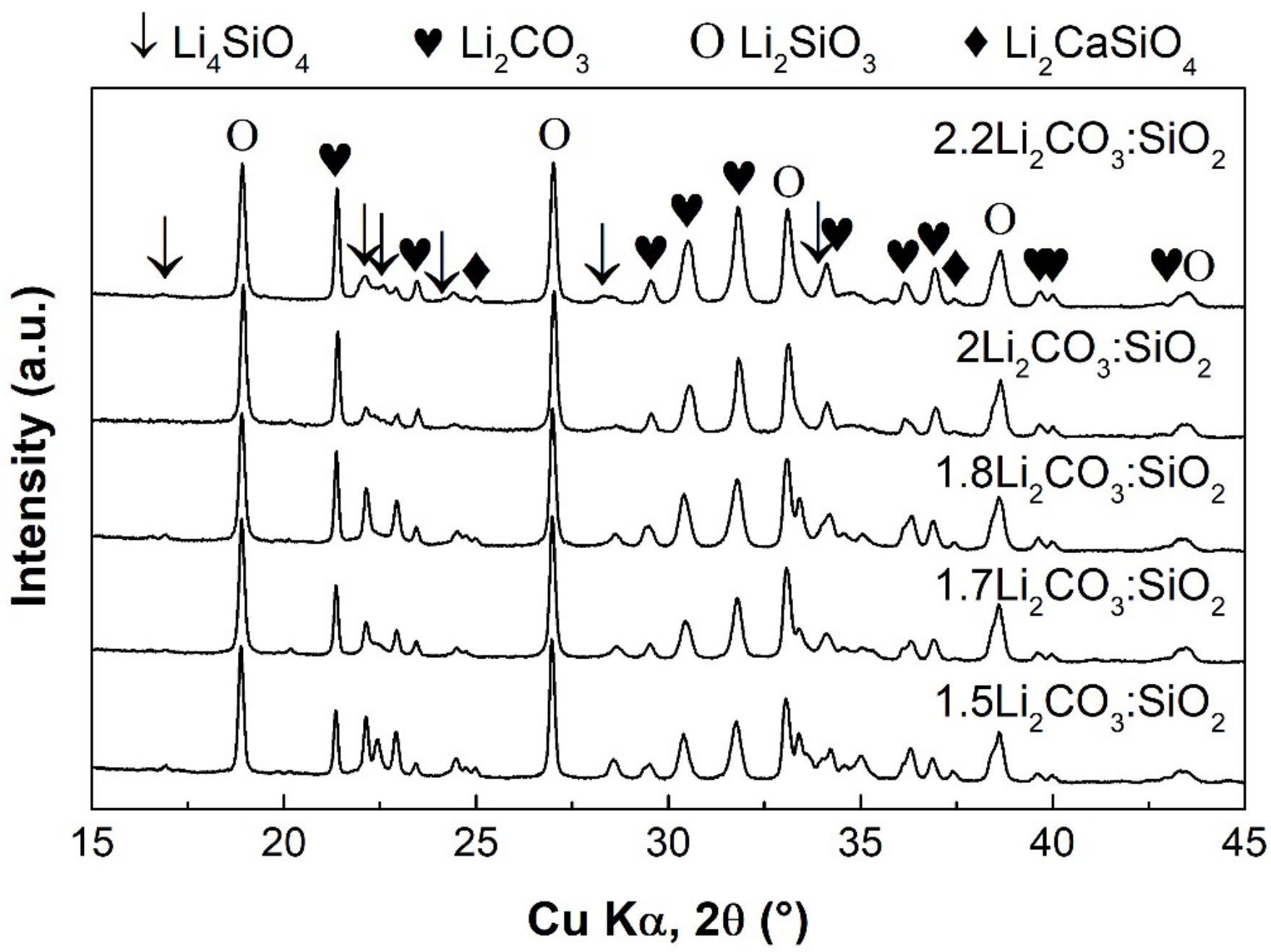

Figure S3. XRD patterns of the Li_4SiO_4 –based sorbents synthesized using different molar ratio of $n$$Li_2CO_3$-$SiO_2$ after carbonation at 650 °C in pure CO_2.

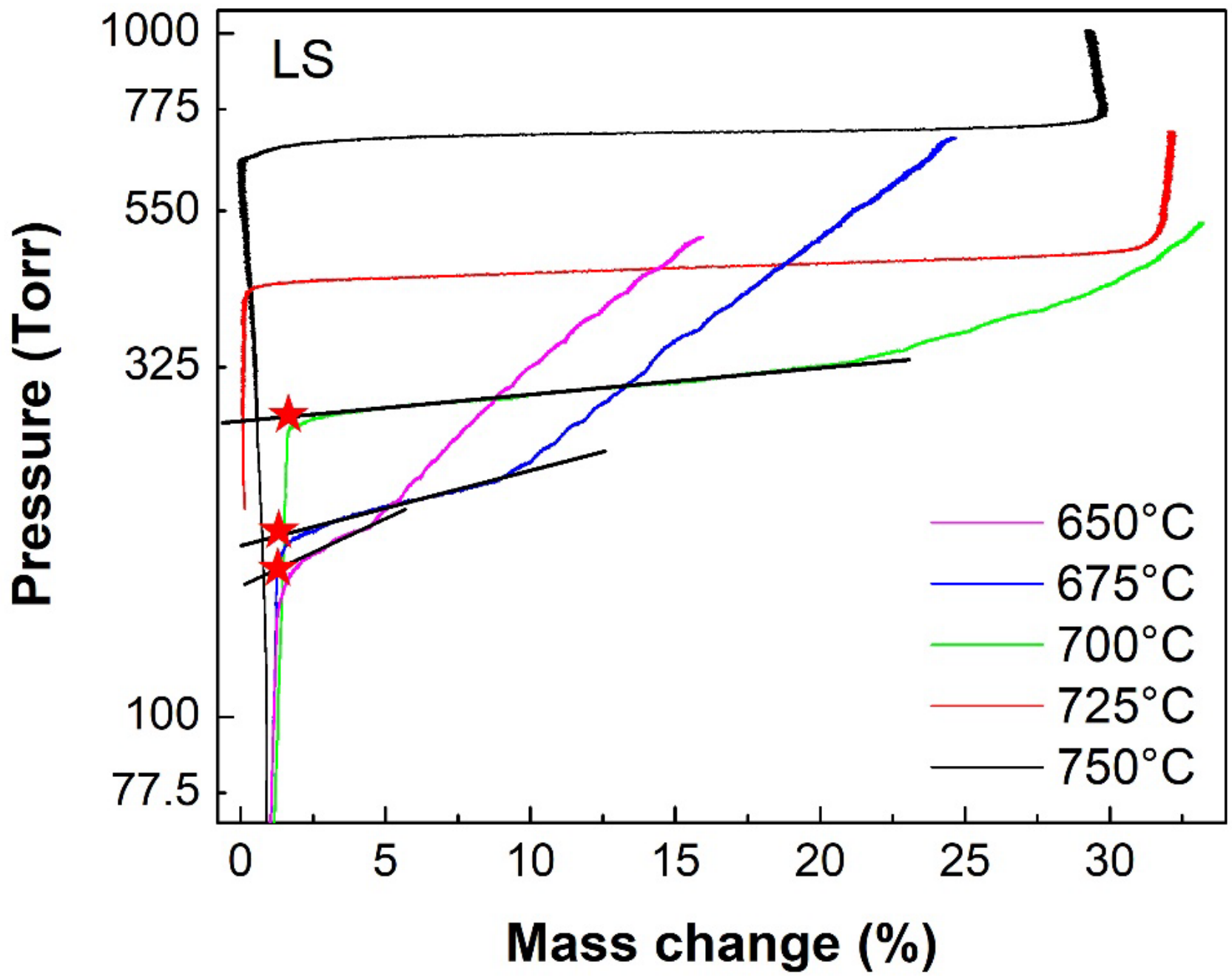

Figure S4. Pressure-composition isotherms (PCIs) for LS carbonation at different temperatures.

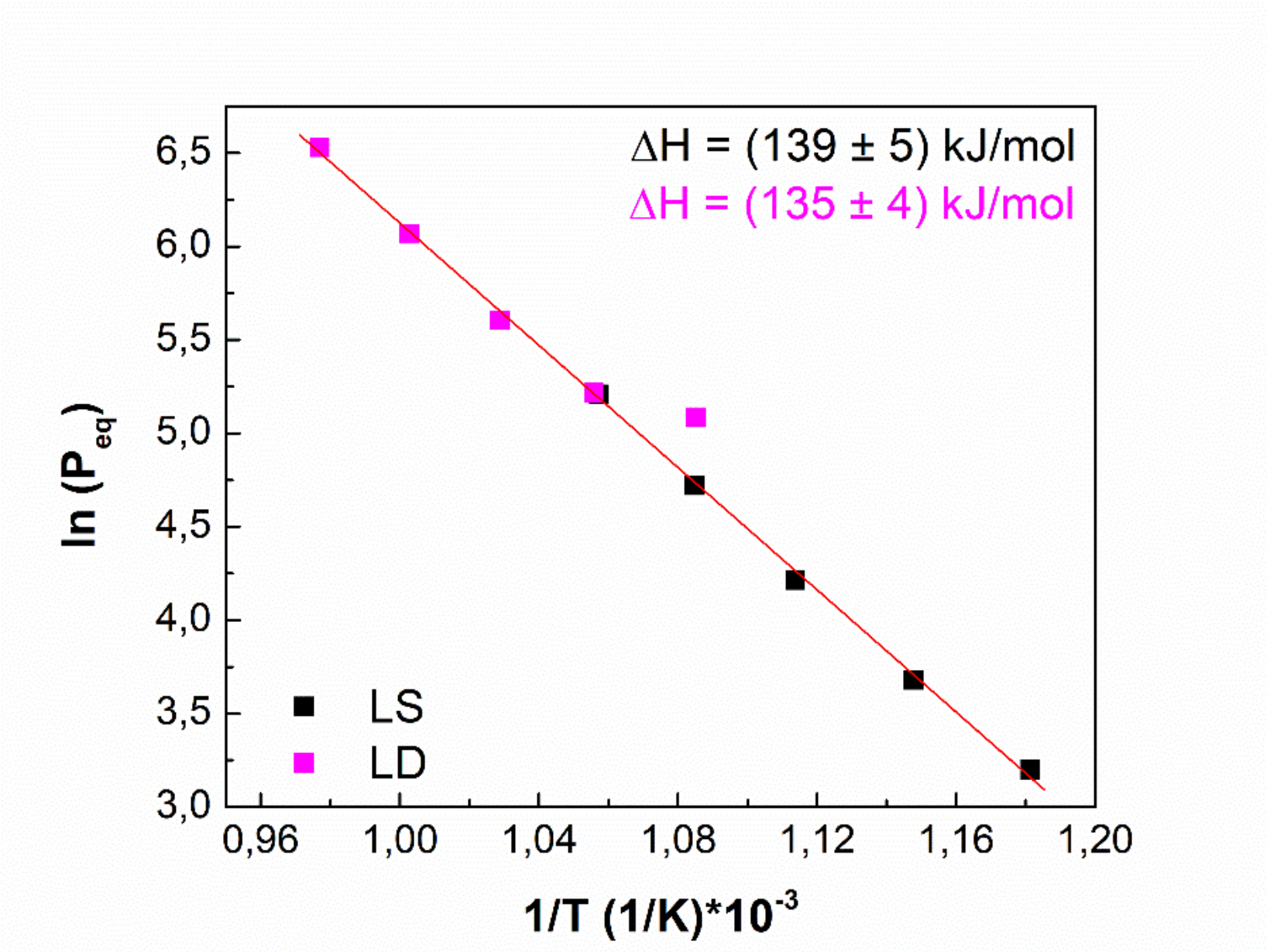

Figure S5. van't Hoff graphs for LD (black dots) and LS (blue dots) derived from equilibrium CO_2 pressures at different temperatures.

Table S1. Equilibrium CO_2 pressures and temperatures determined from pressure-composition isotherms (Figs. 4 and S4), used for calculating the reaction enthalpy via the van 't Hoff equation.

LD	
**Temperature (°C)**	**Equilibrium pressure (torr)**
574	25
598	40
625	68
645	112
673	183
**LS**	
**Temperature (°C)**	**Equilibrium pressure (torr)**
648	161
674	184
699	271
724	431
751	685

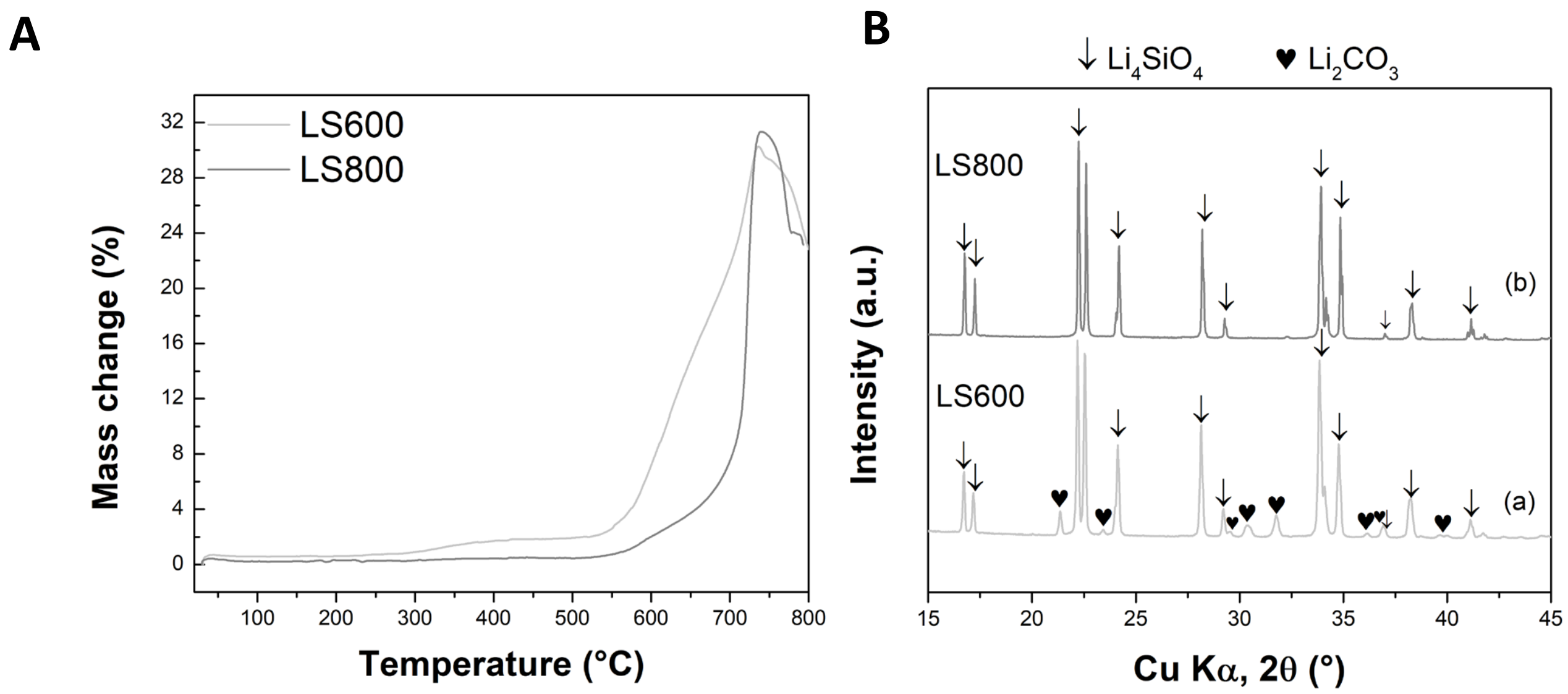

Figure S6. TG curves for LS600 and LS800 under a pure CO_2 flow (A) and their corresponding XRD patterns (B).

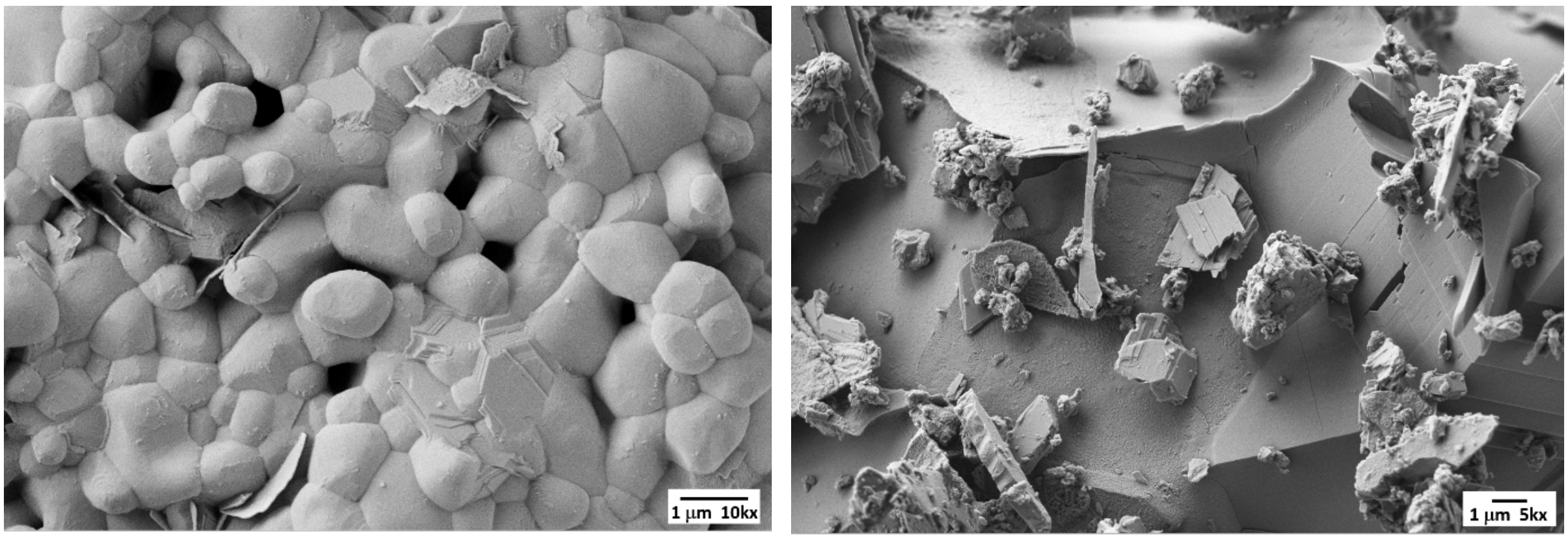

Figure S7. SEM micrographs of Li_4SiO_4 from commercial SiO_2 (A) LS600 and (B) LS800.

Table S2. Crystallite size of Li_4SiO_4 and surface area determined by BET of different samples

**Sample**	**Crystallite size of Li_4SiO_4 (PDF 01-076-1085, 22.2°)***	**BET area (m^2/g)**
Diatomite	-	45
SiO_2 commercial	-	660
LD	22.3° 60 nm	1
LS600	22.2° 83 nm	<1 m^2/g [15]
LS800	22.2° 103 nm	<1 m^2/g [15]

(*) By fitting the XRD data (Fig. 2 R=1.7 and Fig. S6B) and applying the Scherrer equation.

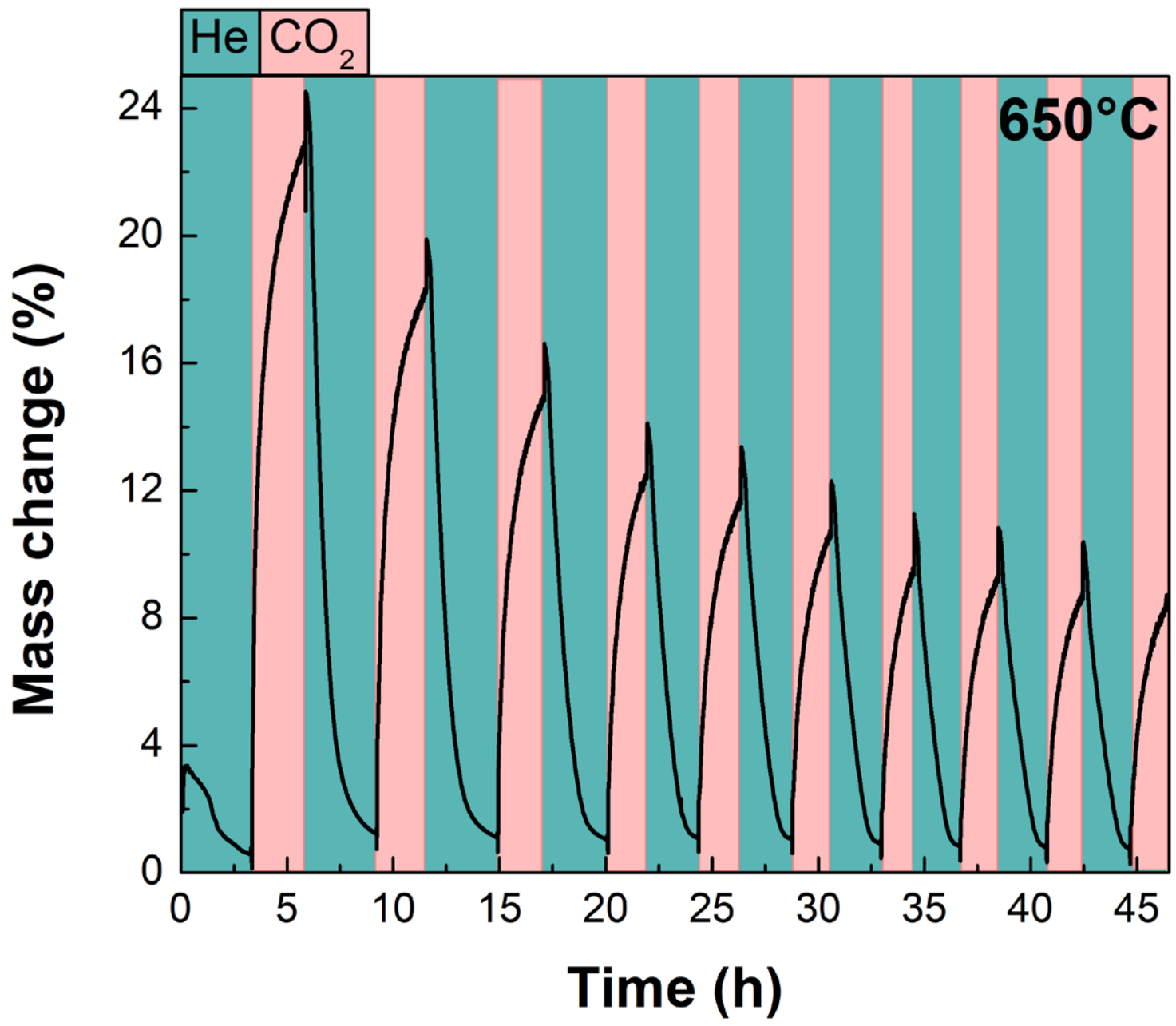

Figure S8. Cyclic CO_2 capture/release performance of the LS600 sorbent over 10 cycles under: pure CO_2/inert atmosphere (at 650 °C).

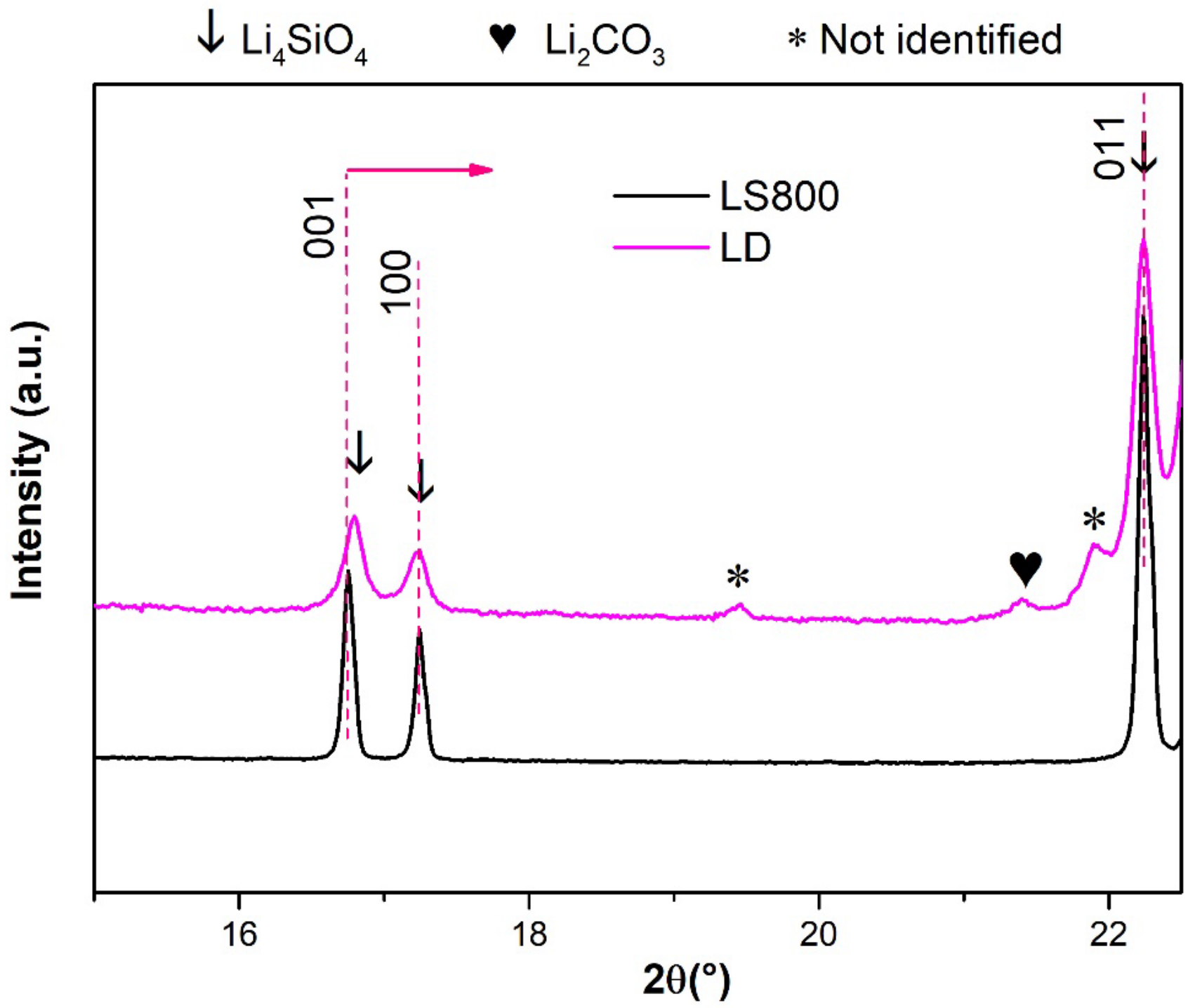

Figure S9. XRD patterns of as-synthesized LD and LS800 in the 2θ range from 15° to 23°.

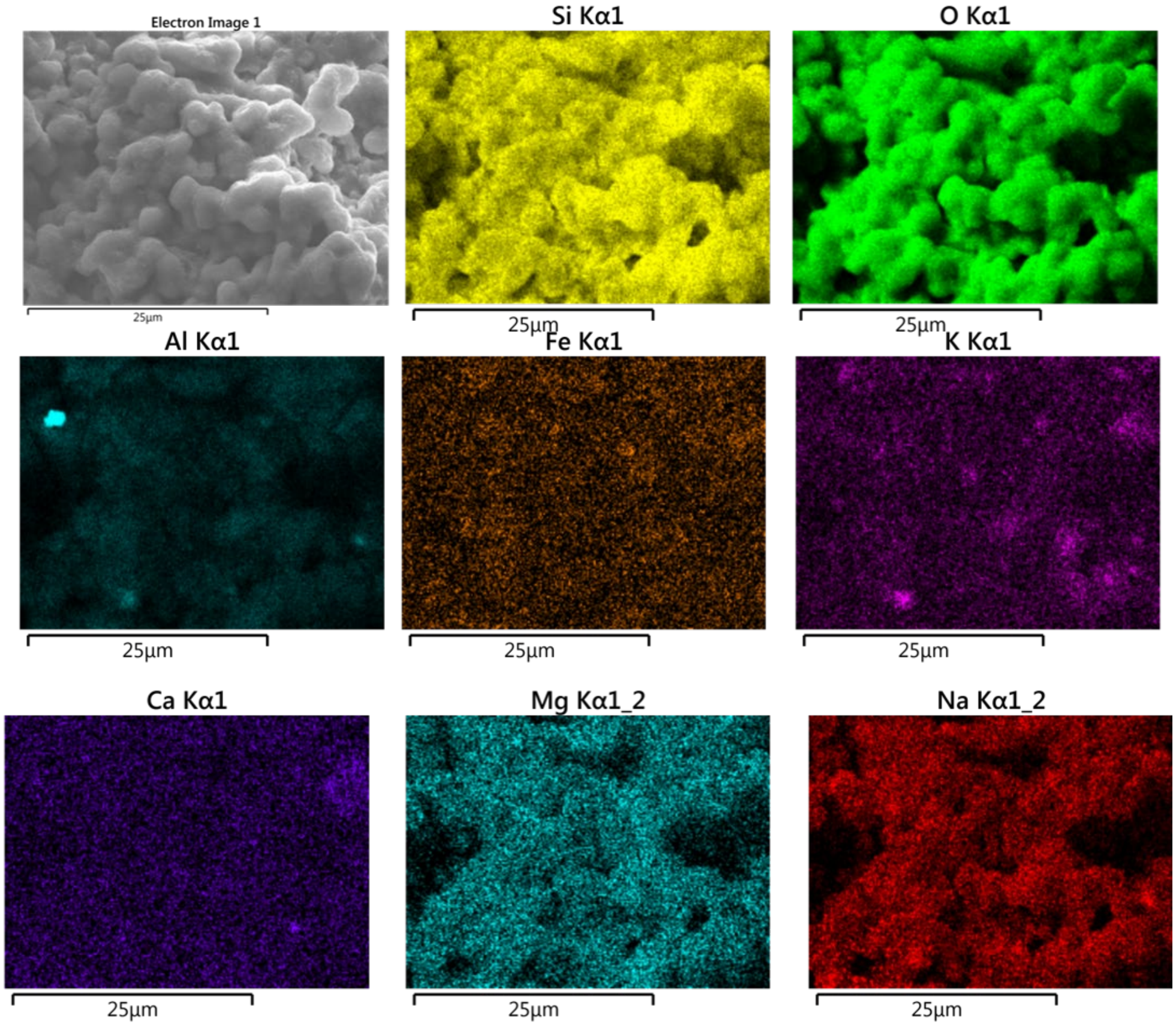

Figure S10. Secondary electron image and chemical mapping of LD after 10 cycles of carbonation/decarbonation under 10% CO_2 at 575° C

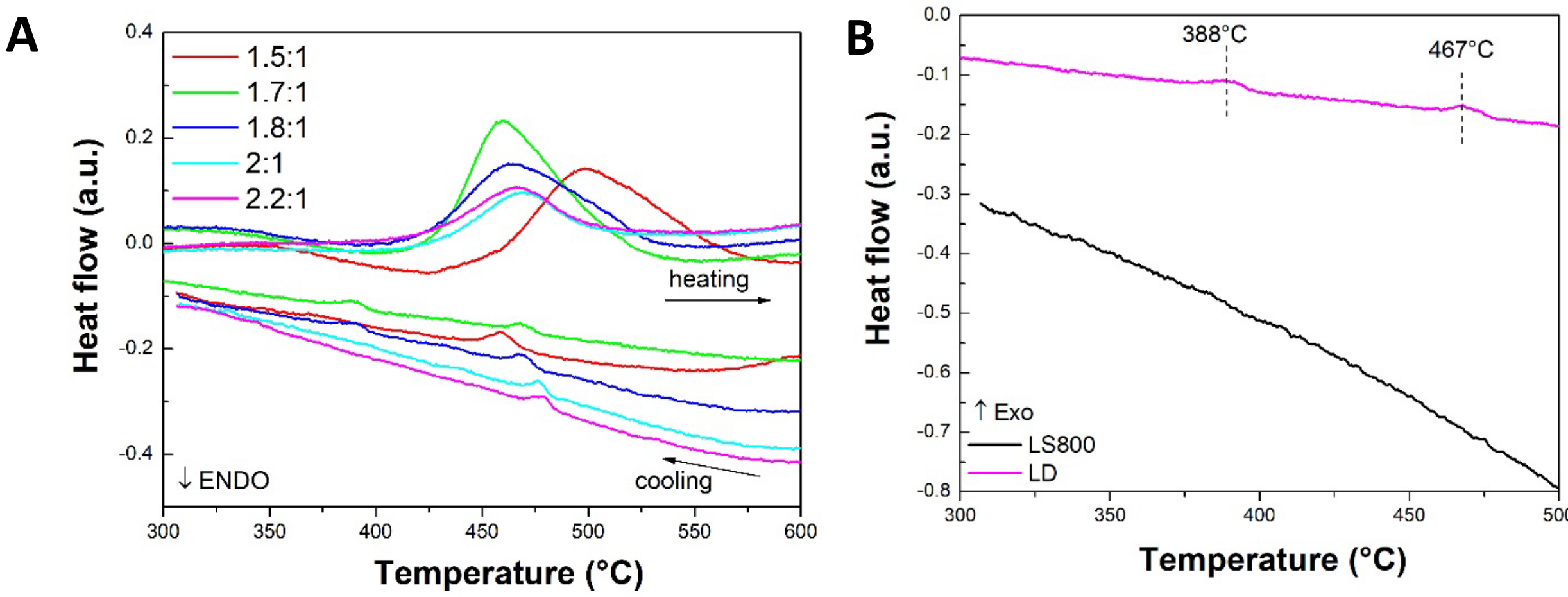

Figure S11. DSC curves obtained during cooling for: (A) LD sorbents with various molar ratios, and (B) comparison between LD (R=1.7) and LS800 sorbents completely carbonated (cooling ramp of 20 °C/min).
